# How the Brain Dynamically Constructs Sentence-Level Meanings From Word-Level Features

**DOI:** 10.3389/frai.2022.733163

**Published:** 2022-04-21

**Authors:** Nora Aguirre-Celis, Risto Miikkulainen

**Affiliations:** ^1^Department of Computer Science, ITESM, Monterrey, Mexico; ^2^Department of Computer Science, The University of Texas in Austin, Austin, TX, United States

**Keywords:** concept representation, embodied cognition, fMRI data analysis, multimodal representation, neural networks, semantic spaces, sentence meaning

## Abstract

How are words connected to the thoughts they help to express? Recent brain imaging studies suggest that word representations are embodied in different neural systems through which the words are experienced. Building on this idea, embodied approaches such as the Concept Attribute Representations (CAR) theory represents concepts as a set of semantic features (attributes) mapped to different brain systems. An intriguing challenge to this theory is that people weigh concept attributes differently based on context, i.e., they construct meaning dynamically according to the combination of concepts that occur in the sentence. This research addresses this challenge through the Context-dEpendent meaning REpresentations in the BRAin (CEREBRA) neural network model. Based on changes in the brain images, CEREBRA quantifies the effect of sentence context on word meanings. Computational experiments demonstrated that words in different contexts have different representations, the changes observed in the concept attributes reveal unique conceptual combinations, and that the new representations are more similar to the other words in the sentence than to the original representations. Behavioral analysis further confirmed that the changes produced by CEREBRA are actionable knowledge that can be used to predict human responses. These experiments constitute a comprehensive evaluation of CEREBRA's context-based representations, showing that CARs can be dynamic and change based on context. Thus, CEREBRA is a useful tool for understanding how word meanings are represented in the brain, providing a framework for future interdisciplinary research on the mental lexicon.

## Introduction

Many experimental studies suggest that there are two types of semantic knowledge: linguistic and experiential (Vigliocco and Vinson, 2007; Vigliocco et al., 2009; Meteyard et al., 2012). Humans acquire linguistic knowledge through a lifetime of linguistic exposure, and experiential knowledge is acquired through their perception and interaction with the physical world. Experiential knowledge denotes the visual, motor, somatosensory, auditory, spatial, cognitive, emotional, and many more attributes of the experienced objects (the referents of words). For example, the word *dog* refers to an entity in the world whose perceived attributes or properties include having four legs, a waggy tail, barks, and so on. Linguistic knowledge includes (spoken and/or written) words defined by their relations to other words in the sentence and in the context in which they are expressed. This knowledge provides individuals with the capacity to communicate about history, scientific terms, ideas, plans, emotions, objects, everything. For example, the word *dog* is defined as a domestic animal, carnivorous, subspecies of the gray wolf, etc.

Further, for each type of semantic knowledge, word meanings arise differently. For linguistic-based knowledge, meaning comes from what people know about the world. For example, for the sentence *He bought the newspaper*, different meanings associated with a single word such as *newspaper* could refer to the printed item bought from a newsstand, or the publishing company. On the other hand, for experiential-based knowledge, meaning comes from the word itself. In this case, there are context-dependent interpretations arising from the same underlying meaning. For example, the meaning of the word *book* for the sentences *The book is heavy*, and *The book is long*, one sentence refers to the weight and the other to the duration in regard to the reader's perception and interaction with a *book*. This is the mental representation of how people perceive and interact with objects, and this is the type of word meaning addressed by this research.

Although humans have a remarkable ability to form new meanings, modeling this process is challenging (Murphy, 1988; Hampton, 1997; Wisniewski, 1997, 1998; Janetzko, 2001; Sag et al., 2001; Middleton et al., 2011). The same concept can be combined to produce different meanings: *corn oil* means oil made of corn, *baby oil* means oil rubbed on babies, and *lamp oil* means oil for lighting lamps (Wisniewski, 1997). Since *lamp* is an object, oil is likely to be a member of the inanimate category. However, *corn* and *baby* are living things, which suggests otherwise. How do language users determine the category membership structure of such combinations of concepts, and how do they deduce their interpretation? As this example illustrates, there are no simple rules e.g., for how *oil* combines with other concepts. Uncovering these mechanisms is the main scientific goal of this paper.

Computational models of such phenomena can potentially shed light into human cognition and advance AI applications that interact with humans via natural language. Such applications need to be able to understand and themselves form novel combinations of concepts. Consider for example virtual assistants such as Siri, OK Google, or Alexa. These applications are built to answer questions in natural language. All of them have natural language processing software to recognize speech and to give a response. However, whereas humans process language at many levels, machines process linguistic data with no inherent meaning (i.e., not connected to the physical world). Their linguistic interactions with users are therefore limited to simple responses. Given the ambiguity and flexibility of human language, modeling human conceptual representations is essential in building AI systems that effectively interact with humans. This is the practical motivation for the work described here.

The work is based on two foundations. The first is a grounding in brain activations. Although early efforts of understanding word meanings were restricted to behavioral observations (Anderson and Ortony, 1975; Potter and Faulconer, 1979; Greenspan, 1986; Medin and Shoben, 1988; Murphy, 1988, 1990; Wisniewski, 1997, 1998), experimental methods have made possible to study the brain mechanisms underlying the semantic memory system. For instance, neuroimaging technology (functional Magnetic Resonance Imaging, or fMRI) provides a way to measure brain activity during word and sentence comprehension. When humans listen or read sentences, they are using several brain systems to simulate seeing the scenes and performing the actions that are described. As a result, parts of the brain that control these actions are activated during the fMRI experiments. Hence, semantic models have become a popular tool for prediction and interpretation of brain activity using fMRI data. This approach will be used in this paper as well.

The second foundation is embodied vector representations. Recently, machine learning systems in vision and language processing have been proposed based on single-word vector spaces. They are able to extract low-level features in order to represent concepts (e.g., cat), but such representations are still shallow and fall short from symbol grounding. In most cases, these models build semantic representations from text corpora, where words that appear in the same context are likely to have similar meanings (Harris, 1970; Landauer and Dumais, 1997; Burgess, 1998; Mikolov et al., 2013; Devlin et al., 2018; Peters et al., 2018). However, such representations lack inherent meaning (Baroni et al., 2014; Erk, 2016; Bender and Koller, 2020), and therefore sometimes even different concepts may appear similar (Andrews et al., 2009; Bruffaerts et al., 2019; Kiefer, 2019; e.g., *night* and *day*). This problem has driven researchers to develop new componential approaches, where concepts are represented by a set of basic features, integrating textual and visual inputs. (Silberer and Lapata, 2012, 2014; Anderson et al., 2013; Silberer et al., 2013, 2017; Bruni et al., 2014; Vinyals et al., 2015). Still, even with these multimodal embedding spaces, such vector representations fall short of symbol grounding. A truly multimodal representation should account for the full array of human senses (Bruni et al., 2014). To meet this challenge, embodiment theories of knowledge representation (Barsalou, 1987, 1999, 2008; Regier, 1996; Landau et al., 1998; Binder et al., 2009) provide a direct analysis in terms of sensory, motor, spatial, temporal, affective, and social experience. Further, these theories can be mapped to brain systems. Recent fMRI studies helped identify a distributed large-scale network of sensory association, multimodal and cognitive regulatory systems linked to the storage and retrieval of conceptual knowledge (Binder et al., 2009). This network was then used as a basis for Concept Attribute Representation (CAR) theory, a semantic model that represents concepts as a set of features that are the basic components of meaning, and grounds them in brain systems (Binder et al., 2009, 2016; Binder and Desai, 2011). Thus, CAR theory will be used to model neural representations of word meaning in this paper.

A particularly intriguing challenge to semantic modeling is that people weigh word attributes differently based on context and recent experiences (Pecher et al., 2004). For example, a pianist would invoke different aspects of the word *piano* depending on whether he will be playing in a concert or moving the *piano*. When thinking about a coming performance, the emphasis will be on the piano's function, including sound and fine hand movements. When moving the piano, the emphasis will be on shape, size, weight, and other larger limb movements (Barclay et al., 1974). The unique focus of this research is to understand this phenomenon, i.e., how word meanings change in the context of a sentence.

The approach is based on the idea that words in different contexts have different representations. Therefore, different features in CARs should be weighted differently depending on context, that is, according to the combination of concepts that occur in the sentence (Anderson and Ortony, 1975; Greenspan, 1986; Medin and Shoben, 1988; Murphy, 1988; Wisniewski, 1997; Potter and Faulconer, 1979). To address this challenge, three central issues on semantic representation are considered: (1) How are concepts represented in the brain? (2) How do word meanings change in the context of a sentence? and (3) What tools can be used to quantify such changes? The first two are addressed using the CAR theory. The approach to the third challenge consists of developing a neural network model called CEREBRA, or Context-dependent mEaning REpresentation in the BRAin, based on CAR theory and constrained by fMRI observations of word meaning. This model is then used to study how the brain constructs sentence-level meanings from word-level features.

Below the CAR theory is first reviewed. After that, sentence and word collections are described, and the CEREBRA framework presented. Computational experiments then demonstrate that (1) words in different contexts have different representations, (2) the changes observed in the concept attributes reveal unique conceptual combinations, and (3) the new representations are more similar to the other words in the sentence than to the original representations. Further, behavioral analysis confirms that the changes produced by CEREBRA are actionable knowledge that can be used to predict human responses.

## The Car Theory

While there are many computational models of word meaning in the literature, most of them fall into two general classes: relation-based, i.e., those in which a word's meaning is represented through its relations to other words (Harris, 1970; Landauer and Dumais, 1997; Burgess, 1998; Mikolov et al., 2013; Devlin et al., 2018; Peters et al., 2018), and feature-based, i.e., those in which it is represented as a set of individual features (attributes). Feature-based models further differ in the way the features are defined, i.e., whether they are abstract (Cree and McRae, 2003; Vigliocco et al., 2004; Vigliocco and Vinson, 2007; McRae and Jones, 2013), or embodied (Binder et al., 2009; Binder and Desai, 2011).

CAR theory (a.k.a. the experiential attribute representation model) is an embodied approach supported by evidence on how humans acquire and learn concepts through sensory-motor, affective, social, and cognitive interactions with the world (Binder et al., 2009; Binder and Desai, 2011). The central axiom of this theory is that conceptual knowledge is built from experience. Particularly, humans learn concepts from birth on through their senses and mental states and these concepts are encoded according to the way they are experienced (e.g., seeing a dog is a visual experience). Since each person's experiences involve different times, locations, cultures, and people, concepts are not static but change throughout lifetime.

In CAR theory, neurobiologically defined “experiential attributes” form a set of primitive features representing the basic components of meaning. This set of features (e.g., Vision, Color, Temperature, Speech, Scene) capture aspects of experience that are central to the acquisition of event and object concepts, both abstract and concrete. The main idea is that people weigh concept features differently based on context, i.e., they construct meaning dynamically according to the combination of words that occur in the sentence (Binder and Desai, 2011). In particular, the features are weighted according to statistical regularities. The semantic content of a given word is estimated from ratings provided by human participants. For example, words referring to things that make sounds (e.g., *explosion, thunder*) receive high ratings on features representing auditory experience (i.e., Loud, Sound), relative to things that do not make a sound (e.g., *milk, flower*).

An important aspect of CAR theory is that its features correspond to the brain systems as listed in Table 1. This approach establishes a connection between conceptual content and neural representations, known as Conceptual Grounding (Harnad, 1990). CAR theory is based on these assumptions: (1) recalling a concept stimulates the features that were active when the concept was first experienced; (2) concepts with similar features produce similar neural patterns; and (3) context modifies the baseline meaning of a concept. The last assumption is the focus of this research. CEREBRA will test such an assumption by characterizing how CARs can be modified to account for the changes in the neural activation pattern of the concept.

**Table 1:** The twelve brain systems and the 66 features used as the basis for the CAR theory.

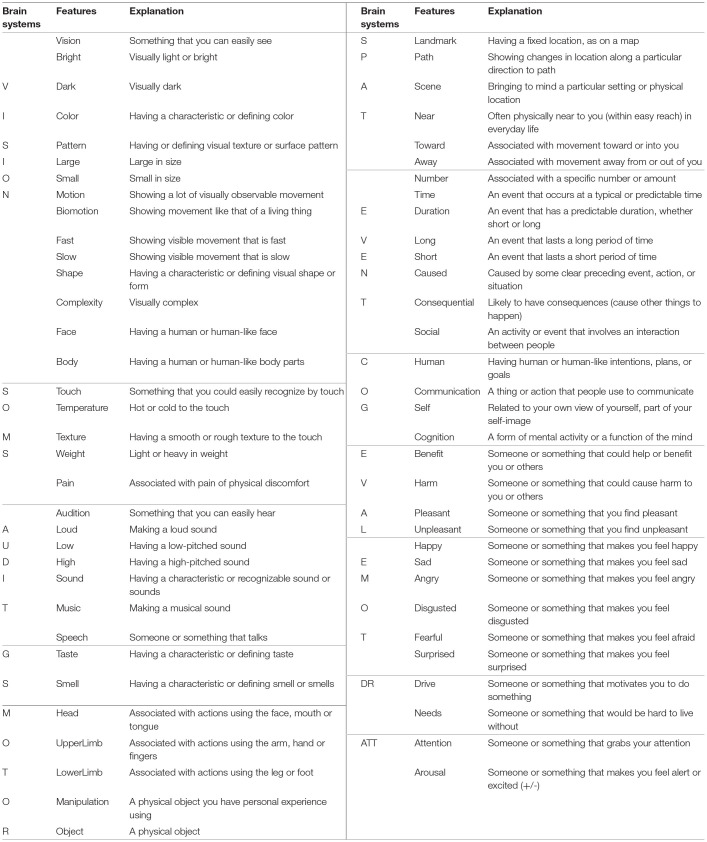

*The first column lists the brain systems. The second column includes the list of features as basic components of meaning. The third column presents a description of each feature. List of attributes representing the semantic system proposed by Binder et al. (2009); Binder and Desai (2011)*.

The terms concept, word and word meaning have specific instantiation in CAR theory, and this instantiation is used throughout this paper. The relation of thought to language is seen as the relation of concepts to meanings. Concepts are seen as a collection of individual features encoded in different neural systems according to the way they are experienced. Words are the symbolic names of concepts, and word meanings are generated when a word is recognized in interaction with its context (Ogden and Richards, 1923). CAR theory thus integrates concepts and word meanings in the same semantic representation. The weights given to the different features of a concept collectively convey the meaning of a word. (Binder et al., 2009, 2016; Binder and Desai, 2011; Binder, 2016; Yee and Thompson-Schill, 2016).

**Figure 1:**
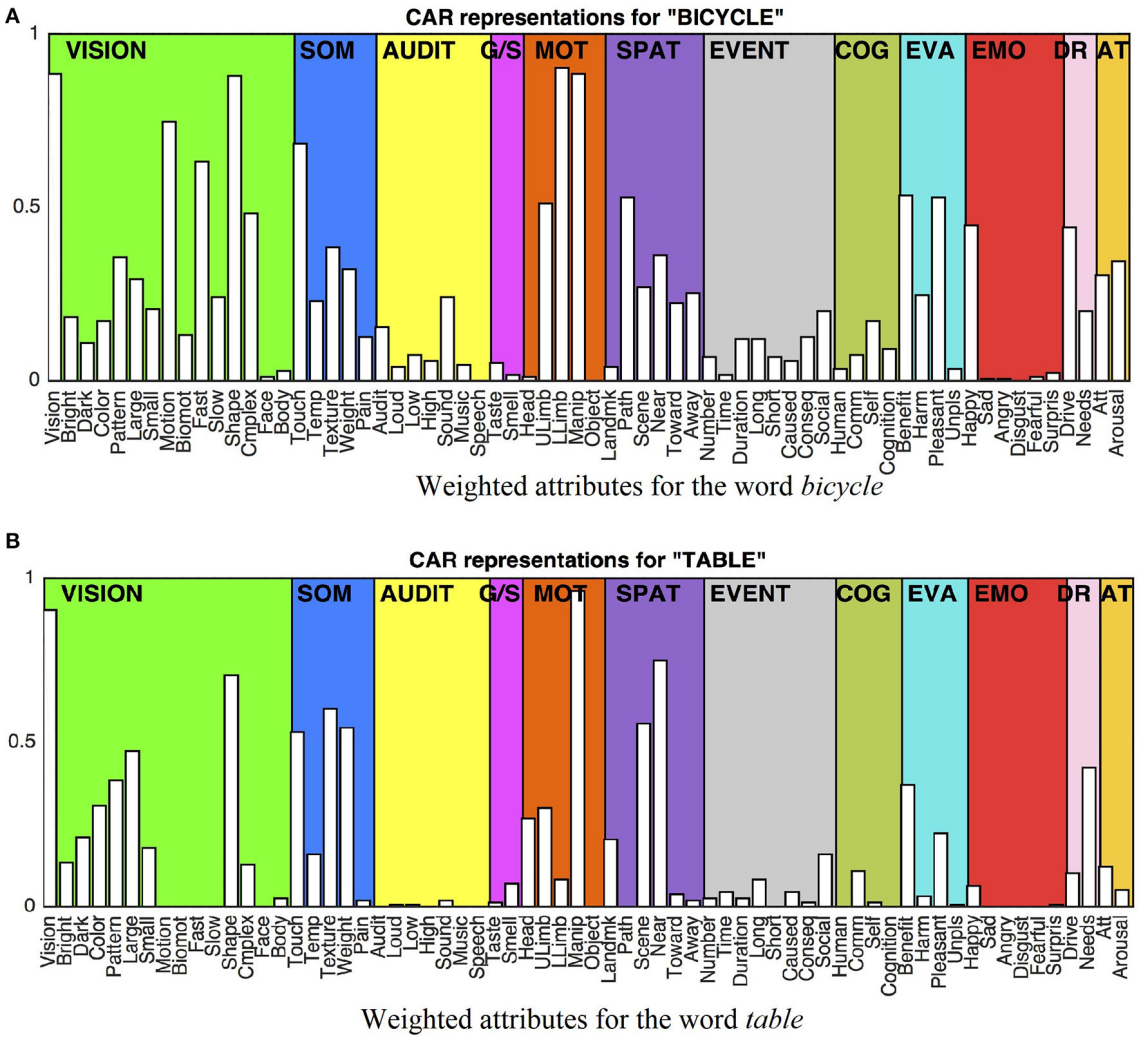
Bar plot of the 66 semantic features for the words *bicycle* and *table* (Binder et al., 2009, 2016; Binder and Desai, 2011). Given that both concepts are objects, they have low weightings on animate attributes such as Face, Body, Speech, Human, and emotions including Sad, and Fear and high weighting on attributes like Vision, Shape, Touch, and Manipulation. However, they also differ in expected ways, including stronger weightings in Motion, Fast, Lower Limb and Path for *bicycle* **(A)** and stronger weightings in Smell, Scene, Near, and Needs for *table* **(B)**. Weighted features for the words *bicycle* and *table*.

More specifically, CAR theory models each concept as a collection of 66 features that captures the strength of association between each neural attribute and word meaning. Specifically, the degree of activation of each attribute associated with the concept can be modified depending on the linguistic context, or combination of words in which the concept occurs. As an example Figure 1, shows the weighted CARs for the concrete concepts *bicycle* (Figure 1A) and *table* (Figure 1B). The weight values represent average human ratings for each feature. Given that both concepts are objects, they get low weighting on animate attributes such as Face, Body, Speech, Human, Communication, and emotions such as Sad, Angry, Disgust and Fear, and high weighting on attributes like Vision, Shape, Touch, and Manipulation. However, they also differ in expected ways, including stronger weightings for *bicycle* on Motion, Biomotion, Fast Motion, Lower Limb and Path, and stronger weightings for *table* on Large, Smell, Head, Scene, Near, and Needs.

In contrast to concrete concepts, abstract concepts refer directly to cognitive events (such as adventure, marriage, future, death), states (such as decide, judge, recall, think), mental “products” of cognition (such as idea, memory, opinion, thought), social cognition (such as justice, liar, promise, trust), and affective states (such as anger, fear, sad, happy, disgust). These concepts are learned in large part by generalization across these cognitive experiences in exactly the same way as concrete concepts are learned through generalization across perceptual and motor experiences (Binder, 2016; Binder et al., 2016).

Concepts can be combined to form new concepts (e.g., *red apple*) and there are general principles that govern such combinations as part of people's world knowledge. Functional groupings known as *ad hoc* categories (Barsalou, 1983), are formed when concepts share the same context-related attribute enhancement. Other types of conceptual combinations illustrate how individual semantic factors allow words to combine. For example, *plastic bottle* is a bottle made out of plastic, but *baby bottle* is for babies.

In CAR theory, conceptual combination occurs when two concepts activate a similar set of brain systems, that is, when their features overlap (attribute congruence). These features are mutually enhanced, altering the similarity between the concepts, and resulting in functional groupings or categorizations. For instance, the difference in meanings for *plastic bottle* vs. *baby bottle* is likely due to the different degree of animacy involved. In the case of *plastic bottle*, Size, Shape, Pattern, Small, Texture, Weight, are activated. In contrast in the case of *baby bottle*, Biological Motion, Face, Body, Head, Taste, Smell, Affective, Social Cognition, are activated, but *bottle* does not activate such systems. Therefore, the meaning of the combination is strongly determined by the degree of attribute congruence. On the other hand, contrary to other language models that are based on word relations to capture word meanings (Harris, 1970; Landauer and Dumais, 1997; Burgess, 1998; Mikolov et al., 2013; Devlin et al., 2018; Peters et al., 2018), CARs cannot capture thematic associations (relations) between words (i.e., *party, celebration, birthday cake, candles, laugh*) unless additional sources provide it (Binder et al., 2009).

Next section discusses the processes and materials used to instantiate the CAR theory through interviews of human subjects. For a more detailed account of feature selection and definition see Binder et al. (2009, 2016) and Binder and Desai (2011).

## Data Collection and Processing

The CEREBRA model is based on the following sets of data: A sentence collection prepared by Glasgow et al. (2016), the semantic vectors (CAR ratings) for the words obtained via Mechanical Turk, and the fMRI images for the sentences, collected both by the Medical College of Wisconsin (Anderson et al., 2016, 2017, 2018, 2019; Binder, 2016; Binder et al., 2016). Additionally, fMRI representations for individual words (called SynthWord) were synthesized by averaging the sentence fMRI (Anderson et al., 2016). Each data set is described next.

### Sentence Collection

This collection was prepared for the fMRI study as part of the Knowledge Representation in Neural Systems (KRNS) project (Glasgow et al., 2016; www.iarpa.gov/index.php/researchprograms/krns), sponsored by the Intelligence Advanced Research Projects Activity (IARPA) under the White House BRAIN Initiative Program (BRAIN Initiative, 2013). The words used in the sentences stand for imaginable and concrete words such as:

#### Objects

Things that exist physically, can be animate or inanimate, natural or man-made. They are often nouns and can be count nouns or mass nouns. Examples: *ball, bicycle, dog, and water*.

#### Actions

Things that are done or experienced by living things. They are often verbs that describe moving, perceiving, feeling, and creating. Examples: *walked, ate, built*, and *drank*.

#### Settings

Locations where or when things happen. They are often nouns that describe indoor or outdoor locations, seasons, and time of day. Examples: *church, forest, spring*, and *morning*.

#### Roles

What people do or who they are. They are often nouns that describe vocations, professions, and kinship. Examples: *banker, doctor, minister*, and *family*.

#### State and Emotions

Descriptive and characterizing words. They are often adjectives that portrays or typifies a noun. Examples: *hot, little, old, red*, and *sad*.

#### Events

Things that take place in space and time, such as human-organized encounters or natural incidents. They are often nouns that describe activities or situations. Examples: *party, flood*, and *hurricane*.

There were a total of 242 such words (141 nouns, 39 adjectives and 62 verbs) in the sentences. A total of 240 sentences were composed from two to five of those words. Sentences are in active voice and consist of a noun phrase followed by a verb phrase in past tense, with no relative clauses. Two hundred of these sentences contain an action verb and the remaining 40 contain the verb *was*. Examples of the sentences include: *The family survived the powerful hurricane, The scientist spoke to the student, The diplomat negotiated at the embassy, The reporter interviewed the politician during the debate, The small church was near the school*.

### CAR Ratings

Binder et al. (2009, 2016), Binder (2016) collected CAR ratings for the original set of 242 words through Amazon Mechanical Turk. In a scale of 0–6, the participants were asked to assign the degree to which a given word is associated to a specific type of neural component of experience (e.g., “To what degree do you think of a *chair* as having a fixed location, as on a map?”). Participants responded by selecting a number where 0 indicates “not at all” and six indicates “very much”. A “Not Applicable” option was also available to cover cases in which the participant felt the question has no logical relation to the word; these responses were coded as 0. Approximately 30 ratings were collected for each word in this manner. After averaging all ratings and removing outliers by rejecting participant responses that had a Pearson‘s correlation coefficient of <0.5 against the mean for that particular word (intraclass correlation; Anderson et al., 2016), the final attributes were transformed to unit length yielding a 66-dimensional feature vector such as those shown in Figure 1 for the words *bicycle* (Figure 1A) and *table* (Figure 1B). The final collection of CAR words consists of 242 word vectors with a 66-dimensional attribute ratings that constitute the generic representation of the words, and is the first essential input to the CEREBRA model: These are the CARWords used as CEREBRA's input (Section Mapping CARs to Synthetic Words).

Note that this semantic feature approach builds its vector representations by mapping the conceptual content of a word (expressed in the questions) to the corresponding neural processes and systems for which the CAR dimensions stand (Binder et al., 2009, 2016; Binder, 2016). This approach thus contrasts with systems where the features are extracted from text corpora and word co-occurrence with no direct association to perceptual grounding (Harris, 1970; Landauer and Dumais, 1997; Burgess, 1998; Baroni et al., 2010).

### Neural Data Collection

If indeed word meaning changes depending on context, it should be possible to see such changes by directly observing brain activity during word and sentence comprehension. In a separate study Binder et al. (2009, 2016), Binder and Desai (2011) identified a large-scale network with individual brain systems involved in the representation of specific attributes of conceptual knowledge (e.g., knowledge of actions, concrete and abstract concepts). Accordingly, Binder and his team collected brain imaging data from several subjects reading the sentences described in Section Sentence Collection, by recording visual, sensory, motor, affective, and other brain systems contained in such a network. The following sections describe the materials and methods used.

#### Neural fMRI Representation of Sentences

The study population consists of 11 healthy, right-handed, monolingual English-speaking adults, aged 20–60, with no history of neurological or psychiatric disorders. Each participant took part in this experiment producing 12 repetitions each.

While in the fMRI scanner, subjects viewed each sentence on a computer screen through a mirror attached to the head coil. To obtain the neural correlates of the 240 sentences, the sentences were presented word-by-word using a rapid serial visual presentation paradigm. More specifically, images of nouns, verbs, adjectives, and prepositions were presented at the same spatial location for 400 ms each, followed by a 200 ms inter-stimulus interval. The mean sentence duration was 2.8 s. Participants were instructed to read the sentences and think about their overall meaning.

The fMRI patterns were acquired with a whole-body Three-Tesla GE 750 scanner at the Center for Imaging Research of the Medical College of Wisconsin (Anderson et al., 2016). The fMRI data were preprocessed using standard methods, including slice timing and head motion correction (AFNI software, Cox, 1996). The most stable, active, and discriminative voxels were then selected, and Principal Component Analysis and zero mean normalization were performed on them.

These transformed brain activation patterns were converted into a single-sentence fMRI representation per participant by taking the voxel-wise mean of all repetitions (Anderson et al., 2016; Binder et al., 2016). The most significant 396 voxels per sentence were then chosen. The size selection mimics six case-role slots of content words consisting of 66 attributes each. The voxels were further scaled to [0.2–0.8]. This collection of 11 subject images for the 240 sentences constitutes the second essential input to the CEREBRA model: These images are the fMRISent target representations required by CEREBRA (Section System Design).

#### Synthetic fMRI Representations of Words

**Figure 2:**
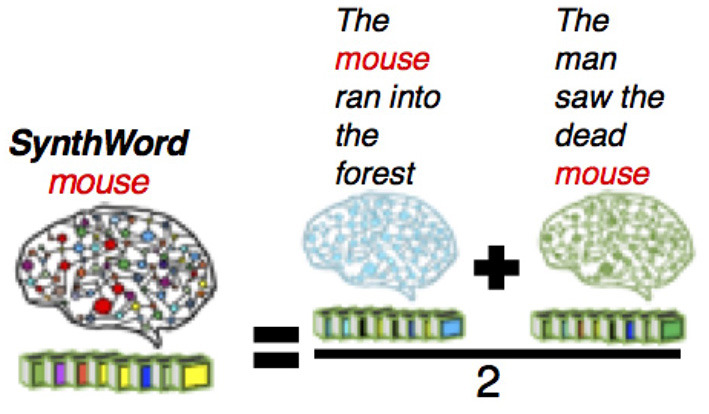
Example of SynthWord representation for the word *mouse* using the average of the two fMRI sentences where the word occurs. SynthWords encode a combination of examples of that word along with other words that appear in the same sentences, that is, the word *mouse* contains aspects of *ran, forest, man, saw*, and *dead*, by averaging the two fMRI sentence representations. SynthWord is derived by averaging the fMRI sentences where the word occurs.

One of CEREBRAs task is to predict fMRI images for words in isolation (described in Section Predicting Sentences and Backpropagating the Error). Unfortunately, the neural data set does not include such images. Therefore, a technique developed by Anderson et al. (2016) was adopted to approximate them. The voxel values for a word were obtained by averaging all fMRI images for the sentences where the word occurs. These vectors, called SynthWords, encode a combination of examples of that word along with other words that appear in the same fMRI sentences. Thus, the SynthWord representation for *mouse* (Figure 2) contains aspects of running, forest, man, seeing, and dead, from the sentences 56:*The mouse ran into the forest* and 60:*The man saw the dead mouse*.

The technique of averaging sentence fMRI is commonly used in imaging studies for that reason (Anderson et al., 2016; Just et al., 2017; Grand et al., 2018). In this case it is specifically supported by neurological evidence suggesting that sentence comprehension consist of a core representation of several word meanings encoded across the brain (Gennari et al., 2007; Anderson et al., 2016).

Due to the limited number of sentences, some SynthWords became identical and were excluded from the dataset. Therefore, the final collection includes 237 sentences and 236 words (138 nouns, 38 adjectives and 60 verbs). This SynthWord collection represents the third essential input to the CEREBRA model: These are the SyntWord representations introduced in System Design.

## Computational Model

CEREBRA uses sentence fMRI patterns (fMRISent; Section Neural fMRI Representation of Sentences) and the CAR semantic feature-based model of concept representations to characterize how word meanings are modulated within the context of a sentence. With CARs of words as input, the neural network is trained to generate initial approximations of fMRI patterns of subjects reading sentences. Then, the FGREP^1^ mechanism (Forming Global Representations with Extended Backpropagation; Miikkulainen and Dyer, 1988) is used to determine how the CARs would have to change to predict the fMRI patterns more accurately. These changes represent the effect of context, and this research aims at characterizing such changes using CEREBRA. It is thus possible to track the brain dynamic meanings of words by tracking how the CARs feature-weightings change across contexts. The following sections describe the computational model and the data that supports it.

### System Design

**Figure 3:**
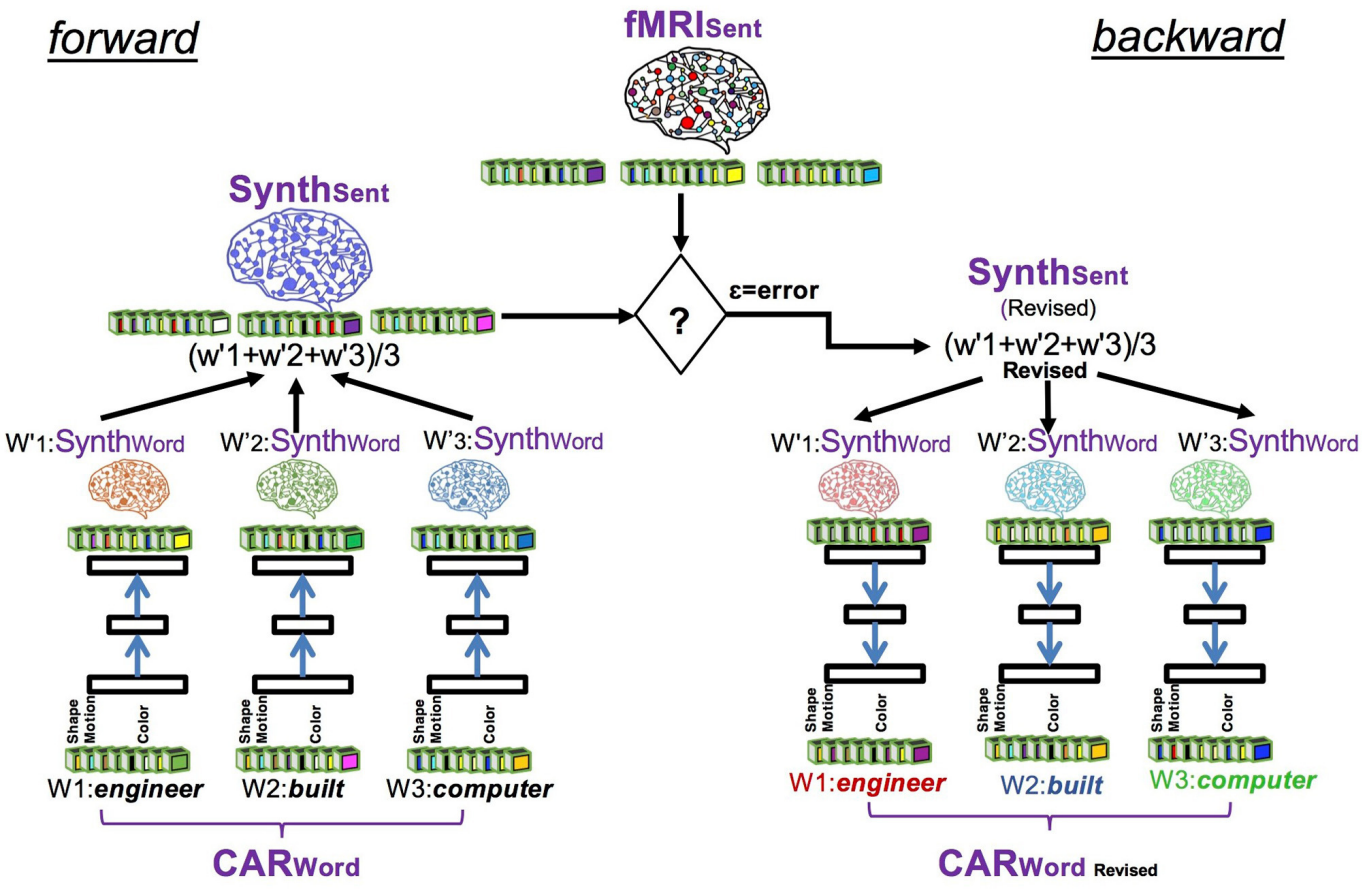
The CEREBRA model to account for context effects. (1) Propagate CARWords to SynthWords. (2) Construct SynthSent by averaging the SynthWords into a prediction of the sentence. (3) Compare SynthSent with the observed fMRI. (4) Backpropagate the error with FGREP for each sentence, freezing network weights and changing only CARWords. (5) Repeat until error reaches zero or CAR components reach their upper or lower limits. The modified CARs represent the word meanings in context. Thus, CEREBRA captures context effects by mapping brain-based semantic representations to fMRI sentence images.

The overall design of CEREBRA is shown in Figure 3. The neural network model serves two main tasks: Prediction and Interpretation. During the Prediction task, the model forms a predicted fMRI for each sentence, without the context effects. Each sentence is thus compared against the observed fMRI sentence to calculate an error signal. This error signal is used repeatedly by the Interpretation task. During the Interpretation task, the model is used to determine how the CARs should adjust to eliminate the remaining error. The error is used to change the inputs (CARs) using Extended-back-propagation (which is the FGREP method). The process iterates until the error goes to zero.

The following sections present a detailed description of the architecture at each stage of the system implementation. CEREBRA is built on several data sets described in detail in Section Data Collection and Processing. Briefly these are: the sentence collection of 237 sentences (Section Sentence Collection), the CAR ratings or semantic representations of 236 words (called CARWord; Section CAR Ratings), the fMRI images of 237 sentences (called fMRISent; Section Neural fMRI Representation of Sentences), and the fMRI synthetic representations for the 236 words (called SynthWord; Section Synthetic fMRI Representations of Words). The specific terms to the CEREBRA model are denoted by abbreviations throughout the paper (e.g., CARWord, fMRISent, SynthWord). For reference, they are described in the Terminology box.

**Figure:**
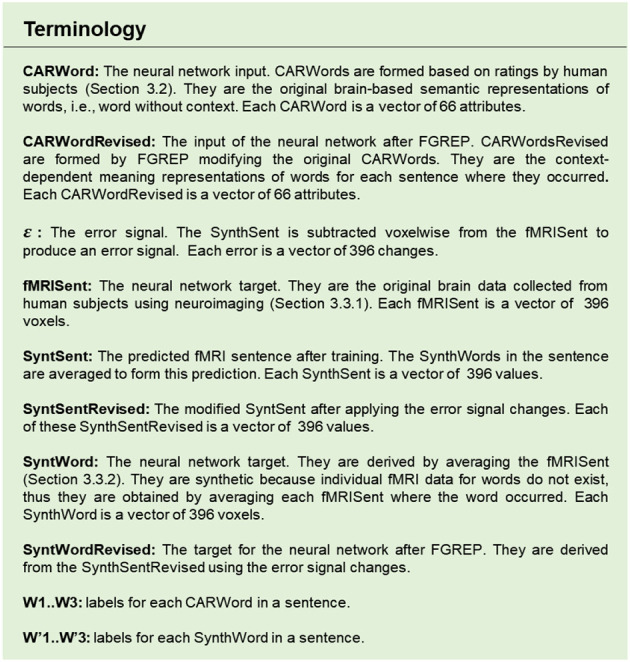


#### Mapping CARs to Synthetic Words

The CEREBRA model is first trained to map the CARWord representations in each sentence to SynthWords (The “forward” side of Figure 3). It uses a standard three-layer backpropagation neural network (BPNN). Gradient descent is performed for each word, changing the connection weights of the network to learn this task (Rumelhart et al., 1986).

Algorithm 1 describes the model implementation and training in detail. A three-layer feed-forward BPNN with 66 input units, 66 hidden units and 396 output units was implemented to map CARs of words to fMRI of words. The training parameters included a learning rate of η = 0.3, decreasing at a rate of 0.001 to 0.000001, to control how quickly the weights will change and avoid converging into a suboptimal solution; and a momentum rate of α = 0.3, to accelerate the training process by helping guide the weights toward the right direction (reducing oscillations). The neural network weighted connections and the bias were randomly initialized between −0.5 and 0.5. The BPNN was trained for each of the 11 fMRI subjects for a total of 20 repetitions each, using different random seeds.

**Algorithm 1:** Neural network to map CAR words to sentence fMRI and back to CARs.

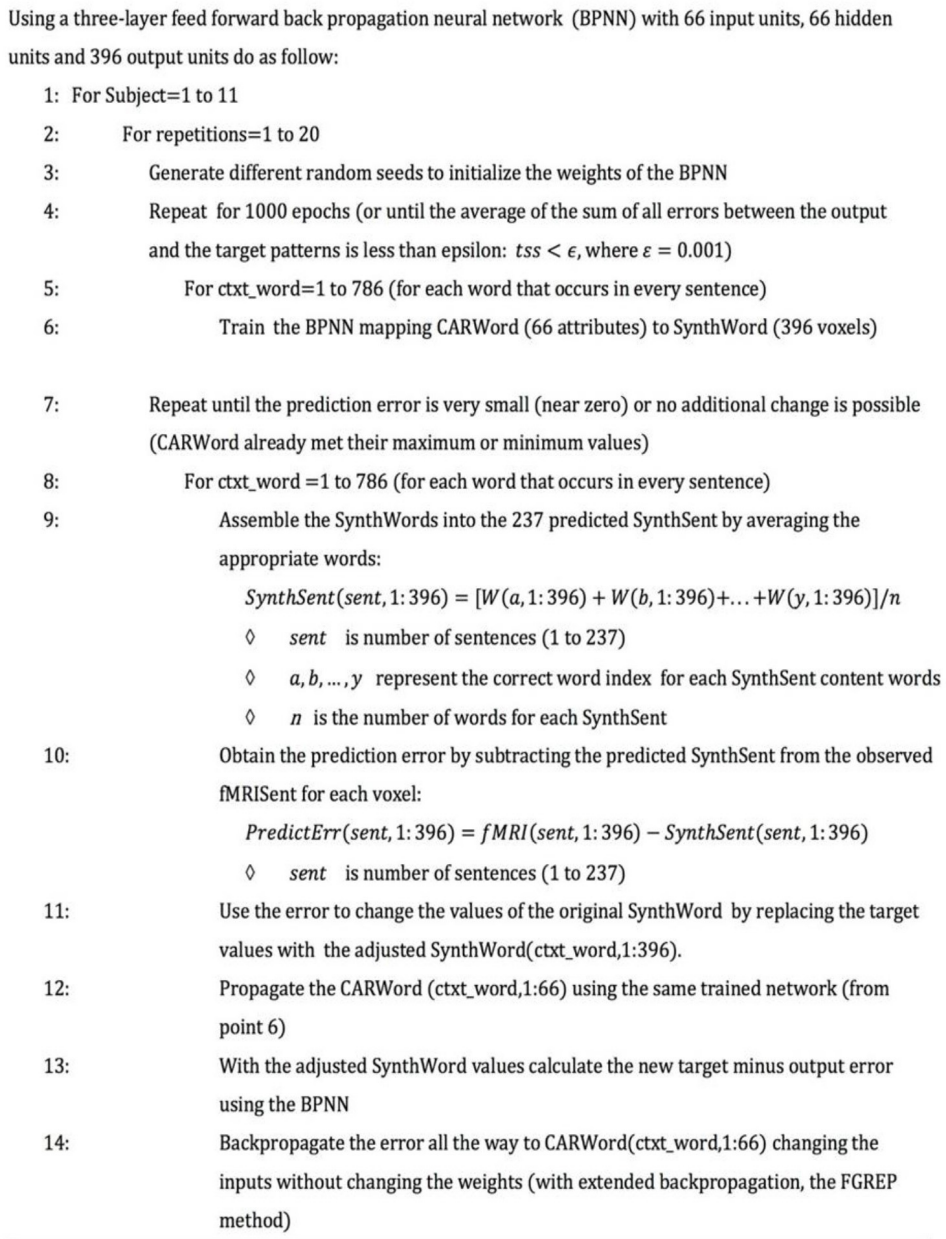

The first part of the algorithm (Step 1 to 6) consists of training the BPNN to map CARWord representations (i.e., input) to SynthWord representations (i.e., target). After training is completed for each subject, it yields 20 different networks, plus 20 sets of 786 predicted SynthWord representations, that is, one word representation for each sentence where the word appears.

#### Predicting Sentences and Backpropagating the Error

The next segment of Algorithm 1 (Steps 7 to 14) describes the Prediction and Interpretation tasks mentioned at the beginning of this section. For the Prediction task, the sentences are assembled using the predicted SynthWords by averaging all the words that occur in the sentence (Step 9), yielding the prediction sentence called SynthSent. For the Interpretation task, in addition to the construction of the predicted sentence, further steps are required (Steps 10 to 14). First, the prediction error is calculated by subtracting the newly constructed predicted SynthSent from the original fMRISent. Then, the error is backpropagated to the inputs CARWords for each sentence (The “backward” side of Figure 3). The weights of the network no longer change. Instead, the error is used to adjust the CARWords in order for the prediction to become accurate.

This process is performed until the prediction error is small (near zero) or cannot be modified (CARWord already met their limits, between 0 and 1), which is possible since FGREP is run separately for each sentence.

As a result, each SynthWord encodes the average meaning of the word. Their combination encodes the expected meaning of the sentence (SynthSent), and the difference of this combination from the actual fMRI encodes the interactions between words. The FGREP modification of the CARs then makes the effects of those interactions explicit. Therefore, the SynthWords do not need to equal the exact fMRI patterns for words in isolation, as long as they carry that information consistently. This is why synthetic fMRI words work well in CEREBRA.

These steps (7 to 14) are repeated 20 times for each subject. At the end, the average of the 20 representations is used to represent each of the 786 context-based words (CARWord Revised), for every single fMRI participant.

Eventually, the Revised CARWord represents the word meaning for the current sentence such that, when combined with other Revised CARWords in the sentence, the estimate of sentence fMRI becomes correct.

### The Role of FGREP Training in CEREBRA

The original FGREP mechanism (Miikkulainen and Dyer, 1988) was designed to (1) learn the processing task by adapting the connection weights using standard backpropagation and (2) develop meaningful distributed representations in the process. In CEREBRA, FGREP is applied in a different manner, and it carries different goals. CEREBRA uses (1) a neural network trained in the task of mapping words from CARWords to SynthWord patterns (Section Mapping CARs to Synthetic Words), and (2) based on an error signal at sentence level, FGREP modifies the baseline meaning of the words (CARWords Revised, Section Predicting Sentences and Backpropagating the Error).

**Figure 4:**
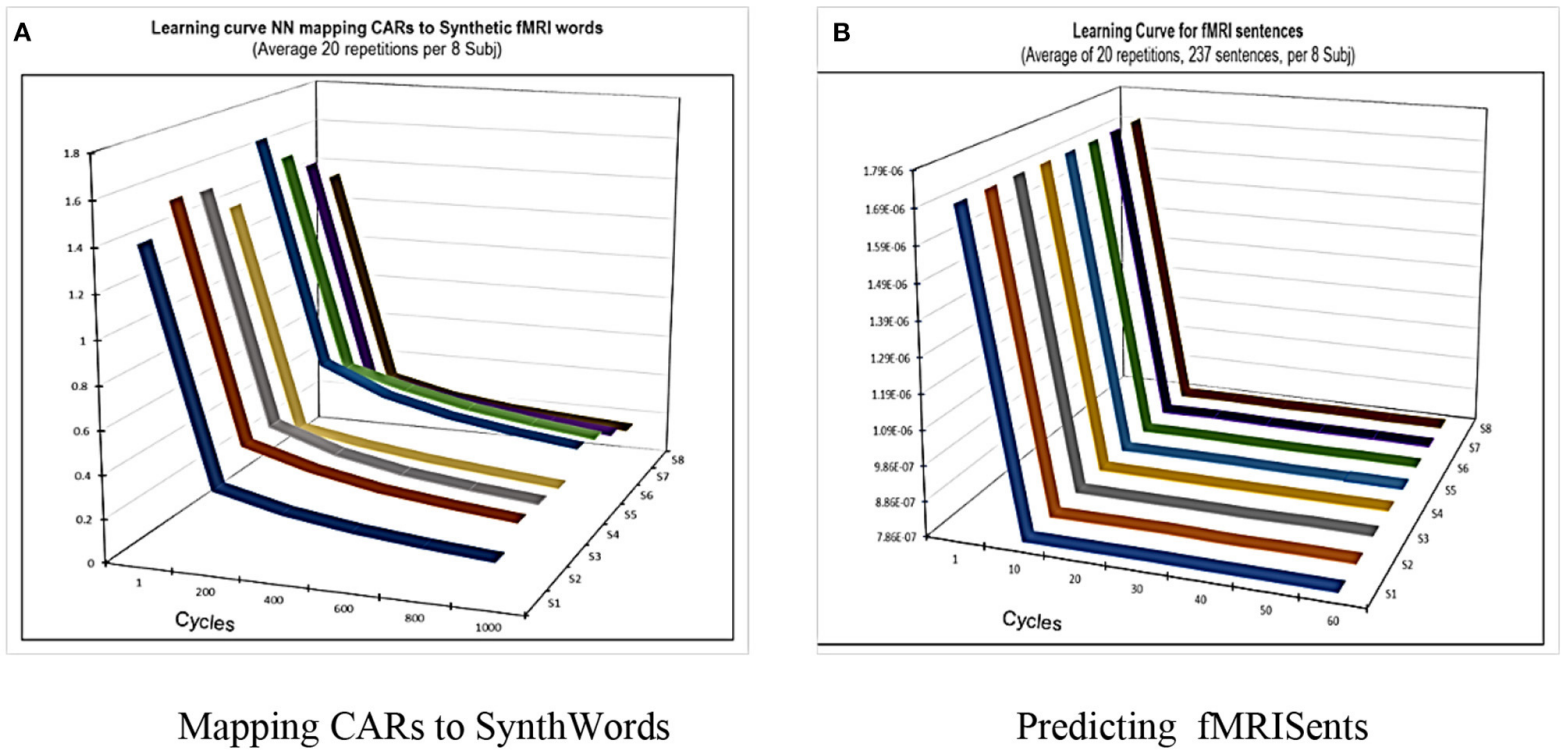
CEREBRA learning performance on the tasks of mapping CARWords to SynthWords and SynthSents to actual fMRISents. The goal of CEREBRA is not to predict the fMRISent patterns as accurately and generally as possible; instead, it is used as a framework to identify and measure context-dependent changes in the CAR words. Nevertheless, it needs to be competent in this task, so these changes are meaningful. The two plots show that it indeed is: **(A)** shows the learning curve on the task of mapping CAR words to Synthetic words. **(B)** shows the learning curve on the task of predicting fMRI sentences. Both are averages over the twenty repetitions for the eight subjects. CEREBRA learns both tasks substantially and quickly.

Therefore, in CEREBRA the neural network is not used in the usual role of achieving general performance in the mapping task (Section Mapping CARs to Synthetic Words). That is, the goal is not simply to predict fMRI sentence patterns accurately and generally; instead, the prediction serves only as a starting point for modifying the CARs. Of course, its performance needs to be competent; the learning curves for each mapping task (SynthWords and fMRISent; Figure 4) demonstrate that indeed it is. Thus, these FGREP networks form a solid starting point for understanding how context affects word meaning.

## Experiments and Results

CEREBRA decomposes sentence fMRI into words and words into embodied brain-based semantic features (CARs). Characterizing how these features change under the context of a sentence, this research will demonstrate that context-dependent meaning representations are embedded in the sentence fMRI, and CAR theory can be used as a foundation for modeling the neural representation of word meaning. The demonstration includes several computational experiments as well as a behavioral study. The computational experiments characterize how the CAR representation of a word changes in different sentences and demonstrates that the linear regression is not powerful enough to capture these changes – a nonlinear model like CEREBRA is needed. The experiments further quantify such changes by correlating them to the CAR representations of the other words in the sentence (OWS) both through individual examples and statistically throughout the dataset. The behavioral study compares CEREBRA's context-based changes to explicit human estimate of those changes, finding that indeed they constitute actionable knowledge.

### Identifying Contrasting Words and Sentences

The Glasgow sentence collection is not fully balanced and systematic, but instead aims to be a natural sample. To investigate the effect of context, finding mutual similarities between words or sentences sounds like a good approach. However, similarity alone is not enough, because anything is similar to anything else to some degree. Contrasting words or sentences is a better mechanism to address such effect. Therefore, a collection of 77 such sentences, with different shades of meaning for verbs, nouns, and adjectives, as well as different contexts for nouns and adjectives was assembled manually (Table 2).

**Table 2:** Collection of 77 contrasting sentences.

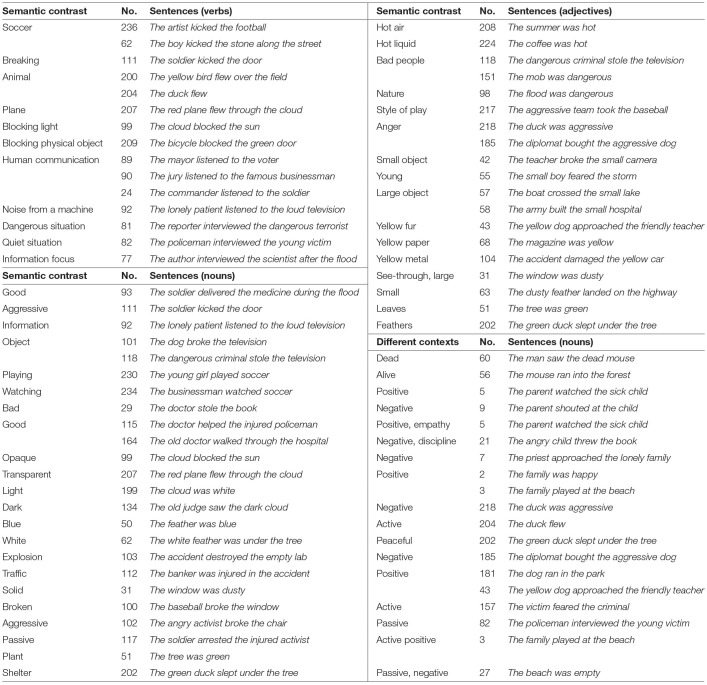

*Sentence examples with differences and similarities in meaning. For instance, the verb kicked in the first two sentences, is used in two different contexts, playing with a ball (as in a soccer game) vs. breaking the door (as an aggressive behavior). Such sentence pairs illustrate the idea of conceptual combination providing the basis for computational models that characterize the effect of context. All content words are target words*.

These sets include differences and similarities like *live mouse* vs. *dead mouse, good soldier* vs. *soldier fighting, built hospital* vs. *damaged hospital*, and *playing soccer* vs. *watching soccer*. Such list allows the computational models to evaluate distinctive attribute representations and consequently adjust the baseline meaning of a word to convey the effects of context and conceptual combination.

Table 2 shows the contrasting sentences. It includes the semantic classification, the sentence number, and the sentence itself. For example, the verb *flew* in sentences 200, 204 and 207 appears in two different contexts: animate (as in *bird* and *duck*) vs. inanimate (as in *plane*). Such contrasting sentences illustrate the idea of conceptual combination and provides the basis for computational models that characterize the effect of context. For this collection, all content words are used as target words for the analyses on the 8 subjects with the most reliable fMRI data (as determined by the fMRI team).

### Multiple Linear Regression

Multiple Linear Regression (LReg) can be used to measure how CARWord change across sentences. If the mapping of semantic representations to fMRI sentences is linear, then LReg will capture such changes. In this section, the LReg approach is described; it will be evaluated in Different Contexts for the Verb “listened”.

Multiple regression is first used to learn the mapping between CARWord and SynthWord voxels at word level. The training set has attribute vectors of words as independent variables and the corresponding SynthWord vectors as the dependent variable, predicting one voxel at the time. Subsequently, at sentence level, the training contains assembled sentences (SynthSent) as the independent variable and the corresponding observed fMRISent as the dependent variable. Once the prediction error is calculated, LReg is inverted (which is possible because it is linear), to determine what the CARWord values should have been to make the error zero.

The Matlab function **fitml** was used to run LReg to map the CARWord to the SynthWord and the inverted linear process to map the SynthWord-revised to produce the CARWord-revised. It uses least squares to predict more than one dependent variable (*Y*) for one or more independent variables (*X*).


$$\begin{array}{l} Y_{i}{\;=\beta_{0\;}+\;\beta_{1\;}X}_{i1}\;+\;\beta_{2\;}X_{i2}\;+\ldots +\beta_{p\;}X_{i2}\;+\;\varepsilon , \end{array}$$

where *i* is the number of observations (depending on the level of process, after 236 words or 237 sentences), *Y*_*i*_ represents the dependent variable, *X*_*i*_ represents the independent variable, β_0_ represents *y*-intercept (constant term), β_*p*_ is the slope coefficient for each independent variable, and ε represents the error or residual.

Additional processes such as assembling the sentences (averaging all words in a sentence) and calculating the predicted and proportional errors were implemented in Matlab scripts.

The experiment in Section Different Contexts for the Verb “listened” tests if the LReg approach and the CEREBRA nonlinear neural network can discriminate between sentences based on feature weightings. The comparison will test whether CEREBRA is a better tool than LReg in bringing out significant changes in word representations.

### Context Effects on Individual Words

This section evaluates experimentally how word meaning changes across different sentence contexts. For conciseness, the experiments presented here analyze example cases where word attributes are weighted differently in various contexts for verbs, adjectives, and nouns (for a comprehensive quantitative analysis, see Aguirre-Celis, 2021).

#### Different Contexts for the Verb “Listened”

**Figure 5:**
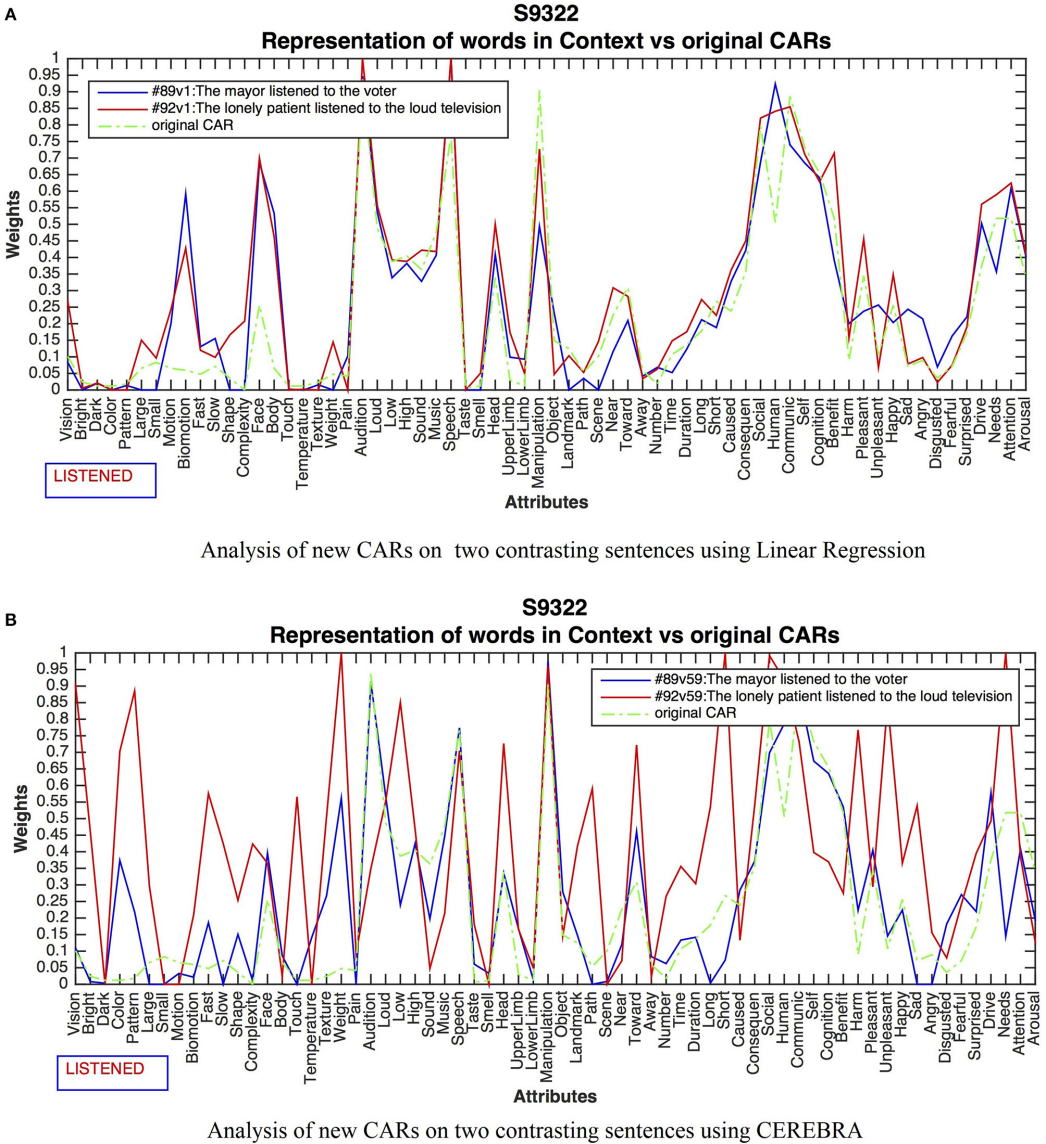
Results for the word *listened* in two contrasting sentences. LReg **(A)** did not capture context. All changes were insignificant to characterizing the context-dependent representations. The green line shows the original CARs for comparison. CEREBRA **(B)** did grasp context. The CARs for Sentence 89 have increased activations in human-related attributes like Face and Body, Auditory attributes, as well as Human, Communication and Cognition. In contrast, Sentence 92 activations on Vision, Color, Large, Shape, Complexity, Touch Temperature, High, Sound, and Unpleasant, depict a loud object such as a television.

This experiment compared the contrasting meanings of HUMAN COMMUNICATION vs. NOISE FROM A MACHINE for the word *listened* as expressed in 89: *The mayor listened to the voter*, 92: *The lonely patient listened to the loud television*. Figure 5A shows the results for LReg between the original and modified CARs for subject 9322. Although the CARs adjusted in all sentences, the changes were small and unprincipled, unable to characterize the difference between human communication vs. noise from a machine. In contrast, the outcome for CEREBRA resulted in context-dependent changes as shown, for sentences 89 and 92 in Figure 5B.

CARs in Sentence 89 presented salient activations in human-related attributes like Face, and Body, Audition, and Speech, as well as Human, Communication, and Cognition, presumably denoting human verbal interaction. For Sentence 92, high activations on Vision, Bright, Color, Pattern, Large, Shape, Complexity, Touch, Temperature, Weight, Scene, Near, Harm, Unpleasant, Happy, and Angry describe a loud and large object such as a television.

These and similar results from other sentences and subjects (Aguirre-Celis, 2021) suggest that the linear mapping that LReg performs is not powerful enough to capture context. A likely explanation is that the relations between the concept attributes and the voxels are too complex to be linearly separable. Indeed, on average the new CAR values with LReg regress to the mean. In contrast, those values in CEREBRA increase, thus gaining new content. The nonlinear mapping provided by CEREBRA is thus powerful enough to capture content, and therefore, subsequent experiments focus on evaluating CEREBRA in this role.

#### Different Contexts for the Adjective “*Dangerous*”

**Figure 6:**
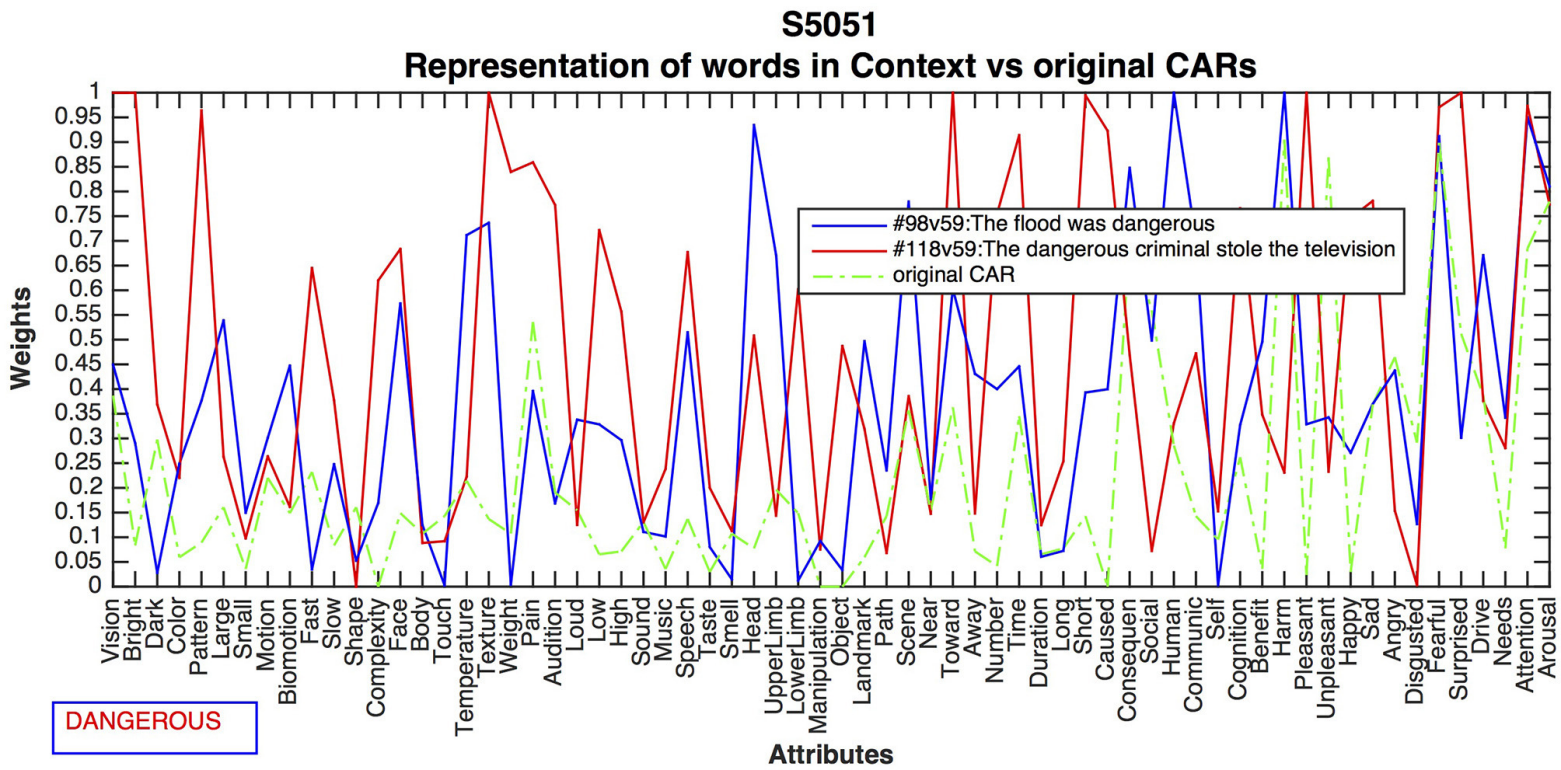
CEREBRA results for the adjective *dangerous* across two contrasting sentences. CARs in Sentence 98 changed activation for Large, Motion, Texture and Weight, Time, Short, and Caused, reflecting moving water. The attributes Toward, Harm, Unpleasant, and Angry, represent the experiential nature of danger. Sentence 118 shows high activation for Vision, Complexity, Face, and Speech, because they represent human types and roles. Lower Limb, Benefit, Angry, Disgusted and Fearful can be associated with a dangerous act by a criminal.

This experiment compared the contrasting meanings of NATURE vs. BAD PEOPLE for the word “dangerous”, as expressed in 98: *The flood was dangerous*, 118: *The dangerous criminal stole the television*. Figure 6 shows the differences resulting from the CEREBRA method for subject 5051. As with the verb *listened*, context-dependent changes did emerge.

CARs in Sentence 98 present changes on activation for Large, Motion, SOMS attributes Texture and Weight, and event attributes Time, Short, and Caused, reflecting moving water. The attributes Toward, Harm, Unpleasant, and the emotion of Angry, represent the experiential and personal nature of danger. Conversely, Sentence 118 shows high activation for Vision, Complexity, Face, and Speech, because they represent human types and roles such as a criminal. Motor attribute Lower Limb as well as evaluation attributes Benefit, Angry, Disgusted, and Fearful can be associated with a dangerous act by a criminal. The CEREBRA method, therefore, was largely able to differentiate between the contrasting relevant dimensions of *dangerous* acts of nature and humans.

#### Different Contexts for the Noun “Mouse”

**Figure 7:**
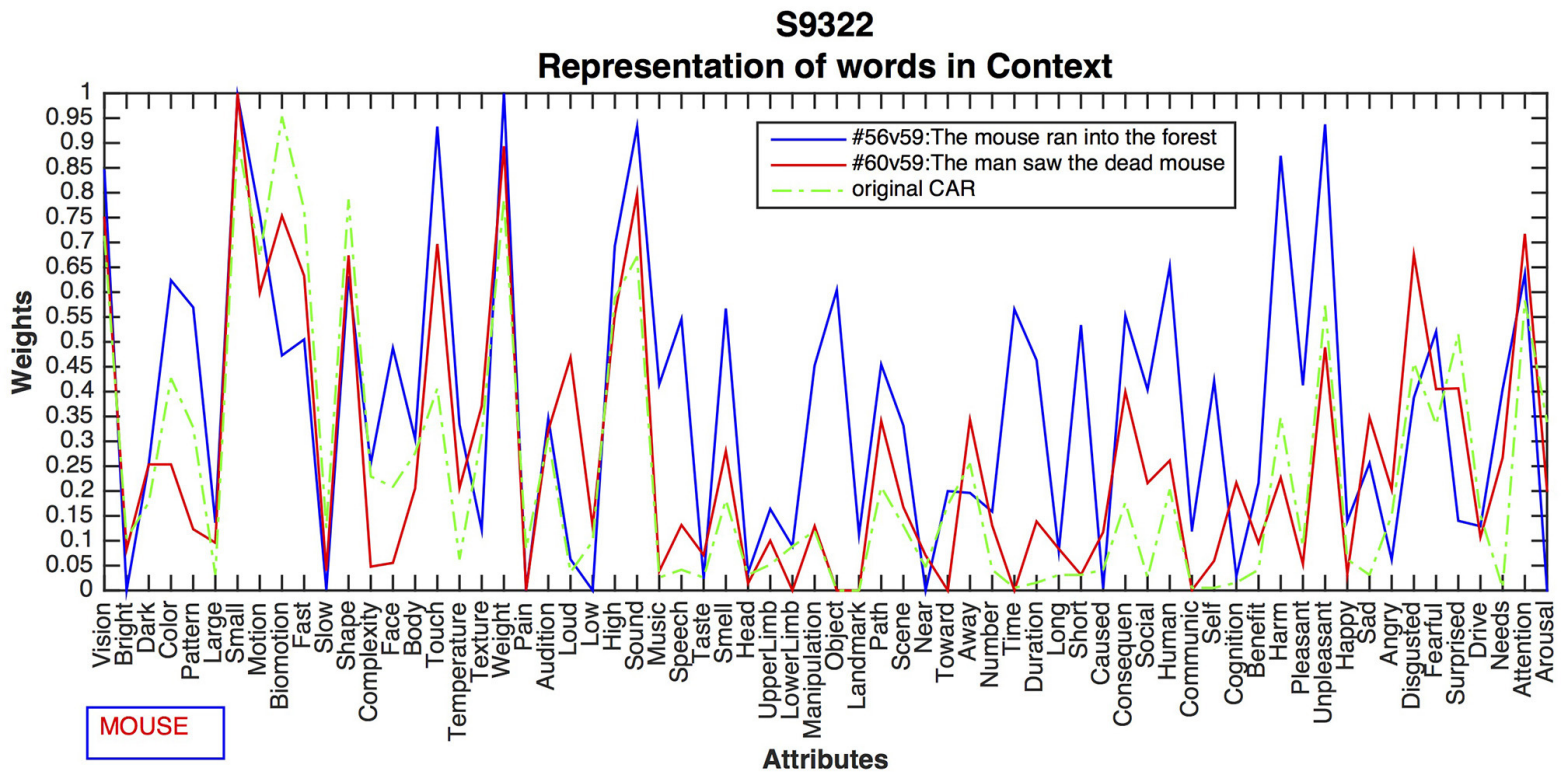
CEREBRA results for the noun *mouse* across two contrasting sentences. CARs in Sentence 56 increased activation for Vision, Motion, Complexity, High, and Sound, presumably to indicate the animate properties of the live mouse. Upper Limb, Path, Away, Time, Duration, Short, and Consequence, suggest activity such as running. In contrast, Sentence 60 shows increased activation for Temperature, Weight, and Smell, as well as Sad, Angry, Disgusted and Fearful, which can be associated with the dead mouse. These changes indicate different aspects of *mouse* in two contrasting contexts.

This experiment compared the contrasting meanings of DEAD vs. ALIVE for the word *mouse* as expressed in sentences 56: *The mouse ran into the forest*, 60: *The man saw the dead mouse*. Figure 7 shows the differences resulting from the CEREBRA method, which are again systematic and meaningful.

CARs in Sentence 56 have increased activation for Vision, Motion, Complexity, High, and Sound, possibly suggesting animate properties of the live mouse. Upper Limb, spatial attributes Path and Away, and event attributes Time, Duration, Short, and Consequence, symbolize activity such as running. Emotions of Fearful and Surprised may well be associated with seeing a live mouse. In contrast, Sentence 60 shows increased activation for Temperature, Weight, and Smell, as well as emotions Sad, Angry, Disgusted and Fearful, which may be associated to the dead mouse. These changes indicate different aspects of *mouse* in two contrasting contexts.

Overall, the results of the experiments in Section Context Effects on Individual Words suggest that different aspects of word meaning are activated in different contexts, and it is possible to see those changes in the corresponding fMRI images using the CEREBRA model. The modified representations in CEREBRA gained content, i.e., they became more descriptive and more distinctive, which provides a good foundation for understanding the structure of the semantic space. In the next set of experiments, the analysis is extended to evaluate the robustness and generality of these conclusions by analyzing combinations of words.

### Conceptual Combination Effect

Earlier work (Aguirre-Celis and Miikkulainen, 2018) showed that (1) words in different contexts have different representations, and (2) these differences are determined by context. These effects were demonstrated by analyzing individual sentence cases across multiple fMRI subjects.

In this experiment, CEBRA analyzes the centrality effect on the attributes of the adjective-noun combinations for the word *small*, as expressed in Sentence 42: *The teacher broke the small camera*, and Sentence 58: *The army built the small hospital*. Centrality expresses the idea that some attributes are true to many different concepts, but they are more important to some concepts than others (Medin and Shoben, 1988). For example, the attribute Small, is more central for a bird than a whale.

**Figure 8:**
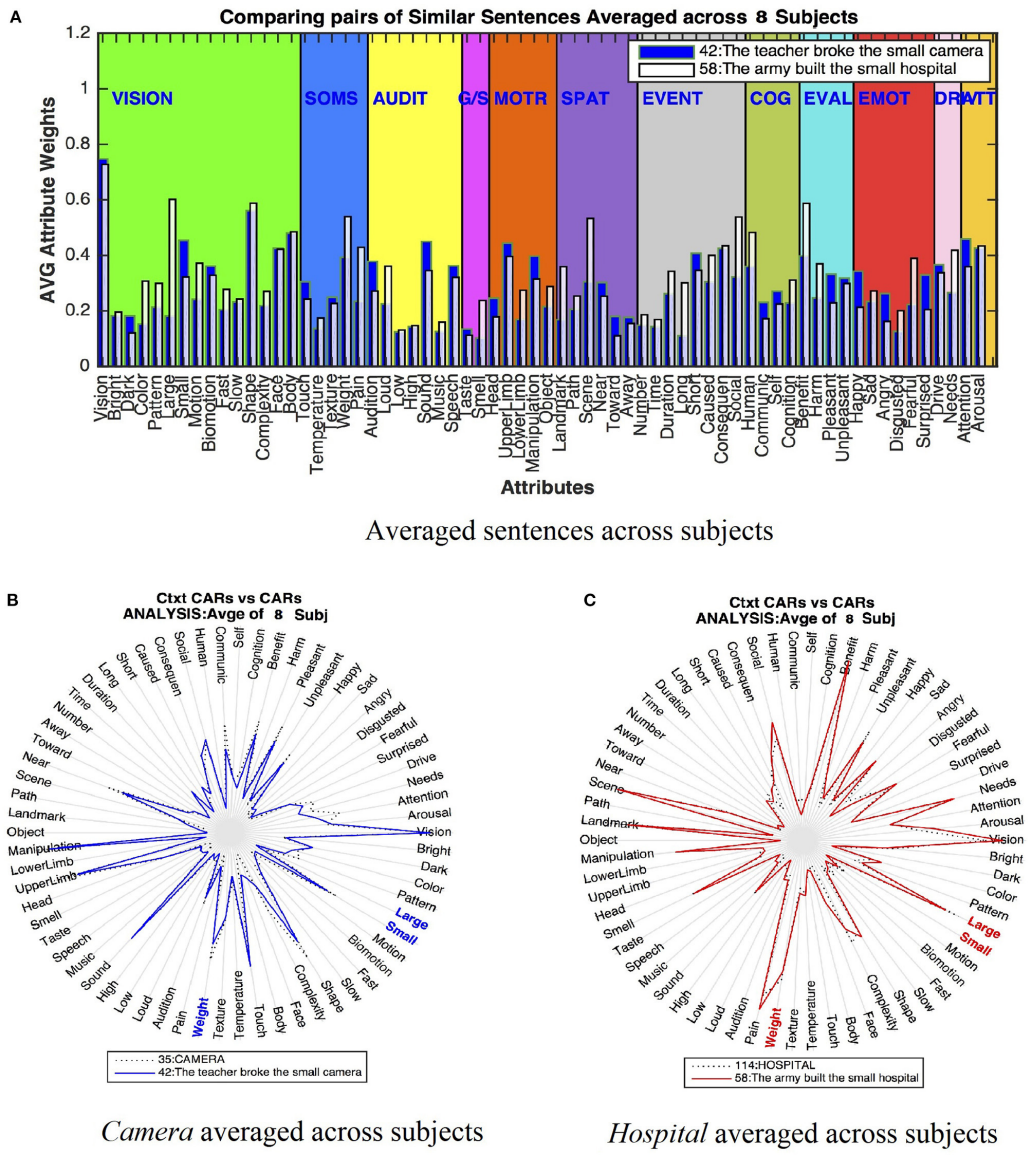
The effect of centrality on two contexts for the word *small*. **(A)** The average for all 8 subjects for the two sentences. Bottom figures **(B,C)** The new *camera* and *hospital* representations averaged for all 8 subjects. The top part **(A)** shows that the new CARs for Sentence 42 have salient activations for an object, denoting the *camera* properties like Dark, Small, Manipulation, Head, Upper Limb, Communication, and emotions such as Sad (e.g., broke the camera). The new CARs for Sentence 58, has high feature activations for large buildings describing a Large, and Heavy structure such as a *hospital*. In the bottom part **(B,C)**, the central attributes are highlighted (Large, Small and Weight) for each word. These emphasize how the same dimensions are more important to some concepts than others. The centrality effect analysis (Medin and Shoben, 1988).

Figure 8 shows the differences for *small* in these two contexts. The top panel (Figure 8A) displays all 66 attributes for the two sentence representations averaged across subjects, and the bottom panel (Figures 8B,C) display the context-based representations averaged across all eight subjects for *camera* and *hospital*.

The size dimensions (e.g., Small and Large), demonstrated the centrality principle for these specific contexts. Figure 8B shows Sentence 42 (e.g., *small camera*) with salient activation for the central attribute Small and low activation for the non-central attribute Large. In contrast, Figure 8C Sentence 57 (e.g., *small hospital*) presents low activation on the non-central attribute Small but high activation on the central attribute Large.

These findings suggest that these attributes are essential to small objects and big structures, respectively. However, the size dimension alone cannot represent the centrality effect completely. This type of conceptual combination requires additional world knowledge to determine the centrality for a particular object, and the relationships between the dimensions of various contexts.

Additionally, given that both *camera* and *hospital* are inanimate objects, Figures 8B,C show how they share low weightings on human-related attributes like Biomotion, Face, Body, and Speech. However, they also differ in expected ways, including salient activations on Darkness, Color, Small and Large size, and Weight. As part of the sentence context, the activations include human-like attributes such as Social, Human, Communication, Pleasant, Happy, Sad, and Fearful. Overall, each sentence representation moves toward their respective sentence context (e.g., *camera* or *hospital*).

These observations are robust and general: analysis was done for all 8 subjects using other types of conceptual combinations (*small bird* vs. *small boy*; *small boy* vs. *small lake*; *bird flew* vs. *plane flew, kicked football* vs. *kicked door*, etc.), producing comparable results.

### Aggregation Analysis

This experiment focuses on the conceptual combination process such as the individual example presented in Section Conceptual Combination Effect. It describes how such a dynamic construction of concepts in the brain can be quantified. This idea was presented anecdotally before, by analyzing a few example cases of how the concept attributes are weighted differently in various sentence contexts. This section expands on this prior work by evaluating the robustness and generality of these conclusions across an entire corpus of sentences and semantic roles (i.e., Agent, Verb, Patient).

The aggregate verifies these conclusions through a statistical analysis: It measures how the CARs of a word change in different sentences and correlates these changes to the CARs of the other words in the sentence. Particularly, it quantifies the conceptual combination effect statistically across sentences and subjects.

The aggregation study hypothesis is based on the idea that similar sentences have a similar effect, and this effect is consistent across all words in the sentence. This effect was verified in the following process (see Aguirre-Celis and Miikkulainen, 2019, 2020a for details):

1. For each subject, modified CARs for each word in each sentence were formed through CEREBRA as described in Figure 2.
2. A representation for each sentence, SynthSent, was assembled by averaging the modified CARs.
3. Agglomerative hierarchical clusters of sentences were formed using the set of SynthSents. The Ward method and Euclidean metric were used to measure the distance between clusters and observations, respectively. The process was stopped at 30 clusters, i.e., at the point where the granularity appeared most meaningful (e.g., sentences describing open locations vs. closed locations).
4. Each cluster of sentences is expected to reveal similar changes in some of the dimensions. To recognize such common patterns of changes, the next step is to calculate the average of the changes for words with similar roles, e.g., *hospital, hotel*, and *embassy* (within the same cluster of sentences). To that end, the differences between the modified and original CAR representations are measured separately for each CAR dimension in each word semantic role, and their significance estimated using Student's *t*-test.
5. The modified CARs of the OWS were averaged.
6. Pearson's correlations were then calculated between the modified CARs and the average CARs of the OWS across all the dimensions.
7. Similarly, correlations were calculated for the original CARs.
8. These two correlations were then compared. If the modified CARs correlate with the CARs of the OWS better than the original CARs, context effect based on conceptual combination is supported.

Specifically, this process aims to demonstrate that changes in a target word CAR originate from the OWS. For example, if the OWS have high values in the CAR dimension for Music, then that dimension in the modified CAR should be higher than in the original CAR for such target word. The correlation analysis measures this effect across the entire corpus. It measures whether the word meaning changes toward the context meaning.

**Figure 9:**
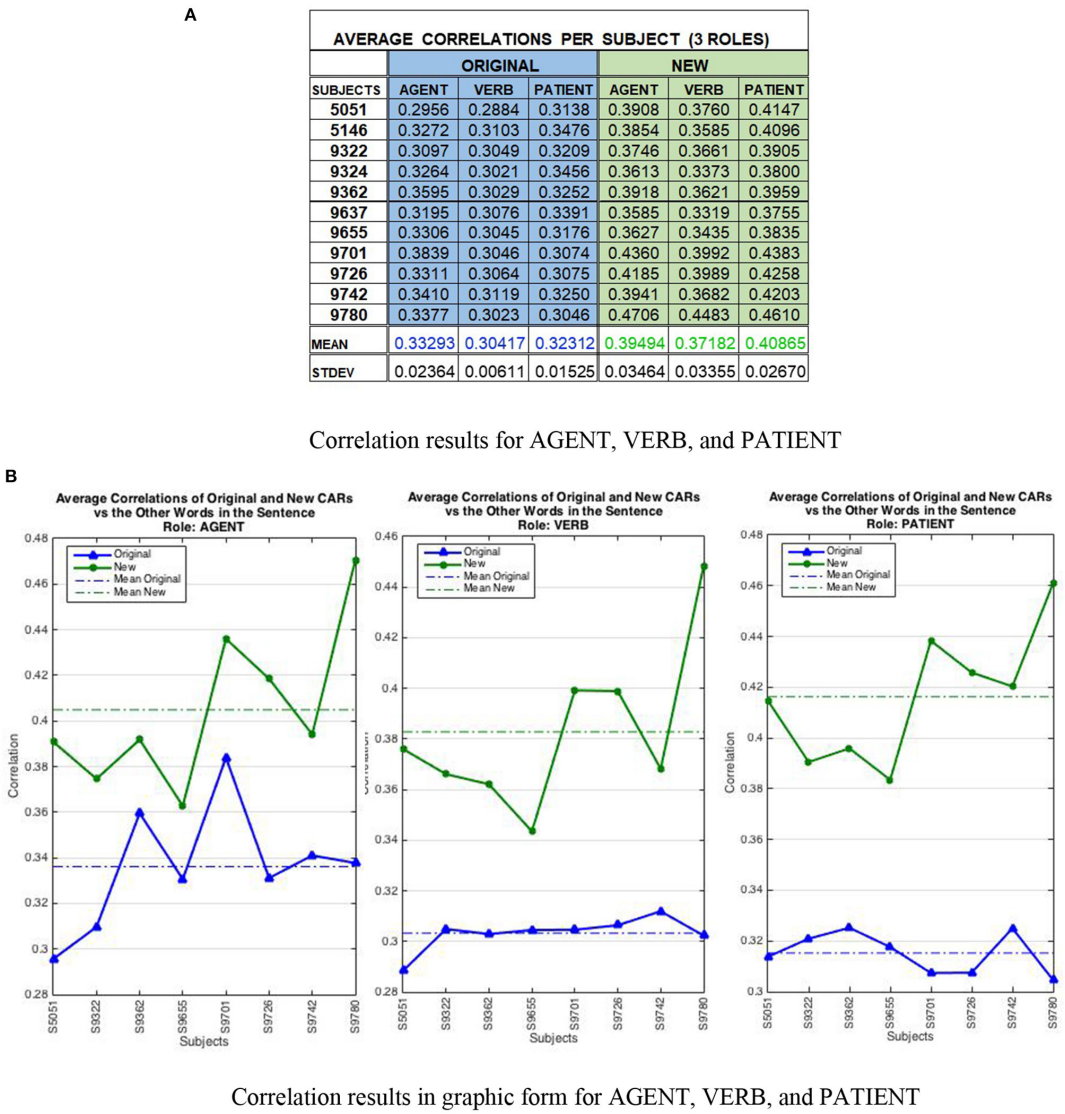
Correlation results per subject cluster. The top part **(A)** displays the correlation data per subject and word semantic role, and the bottom part **(B)** presents the same results in graphic form. The correlations are statistically significant according to the Student's t-test (*p* < 0.05). **(A)** Average correlations analyzed by semantic roles for eight subjects comparing the original and new CARs vs. the average of the other words in the sentence. A moderate to strong positive correlation was found between new CARs and the other words in the sentence suggesting that features on one word are transferred to other words in the sentence during conceptual combination. **(B)** The correlations in graphic form show how the AGENT role represents a large part of the context in both analyses. That is, the original and new patterns are most similar in the AGENT panel, suggesting that this role encodes much of the context. The results show that the conceptual combination effect occurs consistently across subjects and sentences.

The results are shown in Figure 9. The top panel (Figure 9A) presents the correlation results per subject and word semantic roles, and the bottom panel (Figure 9B) displays the results in graphic form. Across all eight subjects and all three semantic roles, the correlations are statistically significant (*p* < 0.05) according to the Student's t-test. Interestingly, the AGENT role represents a large part of the context in both analyses. In other words, the average correlations of the original and modified CARs are most similar in the Agent panel suggesting that this role encodes most of the context. It is important to note that the clusters obtained for each subject's sentences in the aggregation analysis, dictates the way the correlation analysis is conducted for the modified and the original CARs. Each subject produced a different arrangement of sentence clusters that is why the average correlations of the original CARs are different within each role (i.e., they depend on the subject's cluster organization), even though the original CARs include a single set of 236 words compared to the modified CARs that include eight sets of 786 context-based words, or revised CARWords.

Thus, the results indeed confirm that the conceptual combination effect occurs consistently across subjects and sentences, and it is possible to quantify it by analyzing the fMRI images using the CEREBRA model on the CARs. As a summary, the average correlation was 0.3201 (stdev 0.020) for original CARs and 0.3918 (stdev 0.034) for new CARs.

### Behavioral Study

While previous sections have shown that differences in the fMRI patterns in sentence reading can be explained by context-dependent changes in the semantic feature representations of the word, the goal of this section is to show that these changes are meaningful to humans. Therefore, human judgements were compared against CEREBRA predictions (Aguirre-Celis and Miikkulainen, 2020a,b, 2021).

Semantic feature theory suggests that a word meaning is instantiated by weighting its semantic attributes according to the context. (Barclay et al., 1974; Medin and Shoben, 1988; Murphy, 1988, 1990; Hampton, 1997; Wisniewski, 1998; Mitchell and Lapata, 2010; Kiefer and Pulvermüller, 2012; Pulvermüller, 2013). For example, when people think of the word *football*, they heavily weigh features like Shape and Lower Limbs and features like Smell and Size lightly. In contrast, when they think of *forest*, the weighing on those features is likely to reverse. However, when the words appear in the context of a sentence such as *The team lost the football in the forest*, the context might bring up more unusual features like Landmark, Fearful, and Surprise. Thus, when words share features, those aspects of the word representation that are relevant to the context are strengthened (Medin and Shoben, 1988; Murphy, 1990; Hampton, 1997; Wisniewski, 1998; Mitchell and Lapata, 2010; Kiefer and Pulvermüller, 2012; Pulvermüller, 2013).

The hypothesis is that sentence context influences the interpretation of target words by modifying some of their semantic attributes. Consequently, if this attribute changes under the context of a sentence, the fMRI images should embed those changes. Next, the methods and results of the human subject study are described, followed by the methods and results of the computational study. The methods and results of comparing the human judgements and the computational model predictions concludes the study.

#### Measuring Human Judgements

In the survey, subjects were asked to judge how the words change from their generic meaning when they are used in specific sentences. These changes are precisely what the CEREBRA model produces. Thus, the survey made it possible to compare CEREBRA's predictions directly with human judgements.

##### Materials and Design

The survey was constructed to make the comparison as informative as possible based on the fMRI subject data on sentences, words, and attributes.

First, the centroids of each cluster in the aggregation analysis (Section Aggregation Analysis) were selected as the example sentences. They each represent a different context that should have a distinct effect on the words. Across the different subjects, 64 such sentences were found to result in at least 10 statistically significant attribute changes and used for the questionnaire. Second, words in each of the three possible roles of Agent, Verb, and POLE (Patient/Object/Location/Event) were included in each of these sentences, resulting in 38 Agents, 39 Verbs, and 46 POLE words to be tested.

Third, from the 25 attributes with the largest statistically significant change, 10 were randomly selected for each sentence, for four reasons: (1) there is a large number of potentially meaningful attributes, i.e., 25 at least; (2) for simplicity, the survey must not contain many questions; (3) the differences among the top 25 are not considerably large; and (4) it is necessary to get a varied selection of attributes. Choosing the top 10 instead would have resulted in too many visual features for most sentences, either because they frequently changed more, or because visual attributes are more numerous (i.e., 15 out of the 66). The statistically significant attribute changes thus selected represent meaningful differences between the new and the original CAR representations.

**Figure 10:**
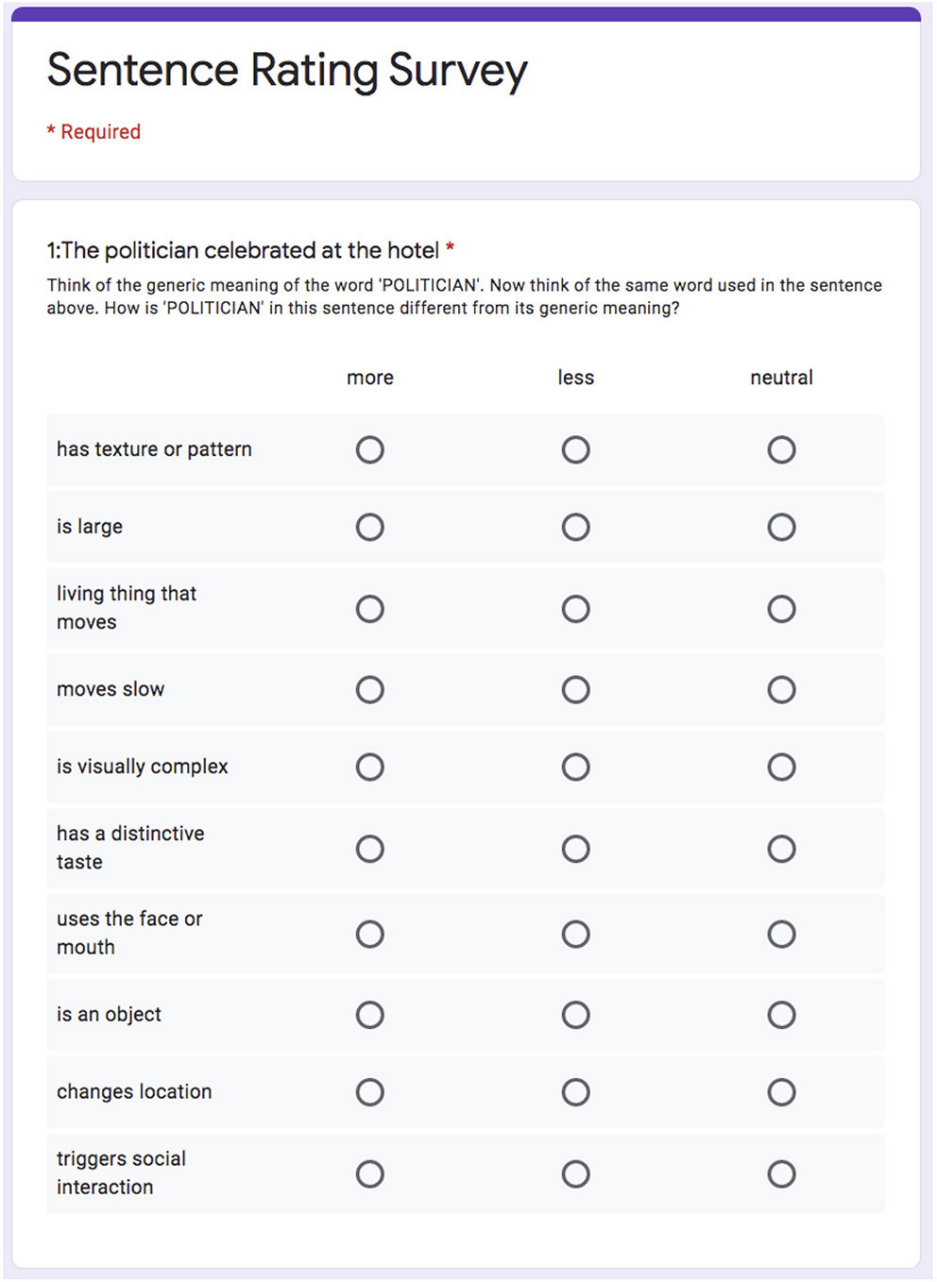
Example sentence in a questionnaire prepared to evaluate the computational model results. The sentence is *The politician celebrated at the hotel*, the target word is *politician* in the role of Agent. Ten different attribute changes are measured by selecting whether the attribute increased (“more”), decreased (“less”) or remained “neutral”. The human judgements were thus matched with those predicted by the CEREBRA model trained with the fMRI data.

To make the questions more understandable for the participants, the original descriptions of the 66 attributes by Binder et al. (2016) were rephrased to make the questionnaires easy to read and to respond to, while retaining their original meaning. The complete survey is an array of 24 questionnaires that include 15 sentences each. For each sentence, the survey measures 10 attribute changes for a particular target word. Overall, each questionnaire thus contains 150 evaluations. For example, a questionnaire might measure changes on 10 specific attributes such as “is visible”, “living thing that moves”, “is identified by sound”, “has a distinctive taste”, for a specific semantic role as in *politician* (Agent), for 15 sentences such as *The politician celebrated at the hotel*. An example sentence questionnaire is shown in Figure 10. Each questionnaire is composed of the Introduction, an Example, and the list of 15 questions.

The entire set of questionnaires can be found at: https://drive.google.com/drive/folders/1jDCqKMuH-SyTxcJ7oJRbr7mYV6WNNEWH.

##### Participants

Human judgements were crowdsourced using Google Forms in accordance with the University of Texas Institutional Review Board (2018-08-0114). The experiments were completed by 27 unpaid volunteers (nine females). The participants' ages ranged from 18 to 64 years, with the mean of 33. Nineteen of them were self-reported bilinguals (English as a second language) and eight English native speakers. Four subjects were affiliated with The University; the rest of the population consisted of working people residing in different parts of north and central America (Texas, Seattle, California, Costa Rica, and Mexico). The subjects had no background in linguistics, psychology, or neurosciences.

##### Procedure

The 24 questionnaires were designed using Google Forms. The respondents were asked to think how the meaning of a specific word changes within the context of a sentence compared to its generic meaning, by evaluating which word attributes change “more”, “less”, or stay the same.

Subjects were recruited by sending emails or text messages directly along with the survey link to access their assigned questionnaire. The data collection was done online, and the participants responded using their cell phone or personal computer. Each questionnaire consisted of an Introduction, Description of the Experiment, Example, and the Survey. Each questionnaire takes about 15 min to complete.

Three of the participants responded to all of the 24 questionnaires. The entire survey consisted of a total of 3,600 questions, so it took them 4–7 days to complete this task at a pace of approximately four questionnaires (i.e., an hour per day). Because this task was a lot of work, the fourth set of responses was obtained by distributing it among multiple raters: 24 additional participants were recruited to each respond to one of the 24 questionnaires.

##### Results

Human responses were first characterized through data distribution analysis. Table 3 shows the number of answers “less” (−1), “neutral” (0), and “more” (1) for each respondent. Columns labeled P1, P2, and P3, show the responses of the three participants that were assigned the entire survey (24 questionnaires, 3,600 answers). Column labeled P4 shows the combined answers of the 24 different participants responding to one questionnaire each. The top part of the table shows the distribution of the rater's responses, and the bottom part shows the level of agreement among them. As can be seen, on average, participants agreed 47% of the time. The Fleiss' Kappa inter-rater analysis revealed that the kappa coefficient (k = 0.202) is statistically significant (*p* < 0.05); indicating the agreement between raters is significantly better than chance.

**Table 3:** Distribution analysis and inter-rater agreement.

**Human responses Distribution**						
**Resp/Part**	**P1**	**P2**	**P3**	**P4**	**AVG**	**%**
−1	2,065	995	645	1,185	1,223	34.0%
0	149	1120	1895	1,270	1,109	30.8%
1	1,386	1485	1060	1,145	1,269	35.3%
TOT	3,600	3600	3600	3,600	3,600	100%
						
**Participant Agreement analysis**						
	**P1**	**P2**	**P3**	**P4**	**Average**	**%**
**P1**	0	1726	1308	1650	1561	43%
**P2**	1726	0	1944	1758	1809	50%
**P3**	1308	1944	0	1741	1664	46%
**P4**	1650	1758	1741	0	1716	48%
				**TOTAL**	**6,751**	
				**AVG xPART**	**1,688**	
		**Average**	Particip match each other			47%
Fleiss-Kappa	Error	Confidence Interval		Agreement	Z	p-value
0.202	0.0048153	0.19955	0.20446	“Fair”	41.951	0

*The top part shows human judgement distribution for the three possible questionnaire responses “less” (−1), “neutral” (0), and “more” (1). The bottom part shows percent agreement for the four raters; Fleiss' Kappa analysis revealed that the agreement between raters is better than chance with a p < 0.05. The task was difficult and the responses noisy. Thus, only the most reliable questions were used to compare to the CEREBRA model*.

Although the inter-rater reliability was low, there were a lot of questions. Thus, it was possible to perform the analysis on those that were reliable enough. In the first such set there were 631 questions where all four participants agreed, or 18% of the total set of questions. In the second such set there were 1,966 questions where at least three of the four participants agreed, or about 55%.

#### Measuring Model Predictions

Three different approaches were designed to quantify the predictions of the CEREBRA model. In order to measure the level of agreement between humans and CEREBRA, a model fitting procedure was implemented.

##### Quantifying the CEREBRA Predictions

The survey directly asks for the direction of change of a specific word attribute in a particular sentence, compared to a generic meaning. Since the changes in the CEREBRA model range within (-1,1), in principle that is exactly what the model produces. However, Aguirre-Celis and Miikkulainen (2019) found that some word attributes always increase and do so more in some contexts than others. This effect is related to conceptual combination (Hampton, 1997; Wisniewski, 1998), contextual modulation (Barclay et al., 1974), or attribute centrality (Medin and Shoben, 1988): the same property is true for two different concepts but more central to one than to the other (e.g., it is more important for boomerangs to be curved than for bananas).

The direction of change is therefore not a good predictor of human responses; instead, these changes need to be measured relative to changes in the other words. Thus, the problem was addressed by three different approaches:

1. What is the effect of the rest of the sentence in the target word? This effect was measured by computing the average of the CEREBRA changes (i.e., new-original) of the other words in the sentence and subtracting that average change from the change of the target word.
2. What is the effect of the entire sentence in the target word? This effect was measured by computing the average of the CEREBRA changes (i.e., new-original) of all the words in the sentence including the target word and subtracting that average change from the change of the target word.
3. What is the effect of CARs used in context as opposed to CARs used in isolation? This effect was measured by computing the average of the CEREBRA changes (i.e., new-original) of the different representations of the same word in several contexts and subtracting that average change from the change of the target word.

The first two approaches have the advantage of being simple. However, the third approach is motivated by neurological evidence suggesting that sentence comprehension involves a common core representation of multiple word meanings combined into a network of regions distributed across the brain (Gennari et al., 2007; Anderson et al., 2016). In line with this view, a generic (or isolated) word representation can be formed by averaging the activity in multiple sentence contexts.

In each of these cases, the resulting vectors are expected to accurately represent the direction of change asked in the questionnaires. They are the ratings used in the evaluation procedure described in the following section.

##### Procedure

Starting from a different random seed, the CEREBRA model was trained 20 times for each of the eight best fMRI subjects (i.e., where the fMRI data in general was most consistent). Responses for each model were thus obtained for the 631 questions where all four subjects agreed, and for the 1,966 questions where three out of four agreed. In order to demonstrate that the CEREBRA model has captured human performance, the agreements of the CEREBRA changes and human surveys need to be at least above chance. Therefore, a baseline model that generated random changes in the same range as the CEREBRA model was created. The chance model was queried 20 times for each of the 631 questions and for the 1,966 questions, for each of the eight subjects. In this manner, 20 means and variances for each of the eight subjects for both CEREBRA and chance were created.

To estimate the level of agreement of CEREBRA and chance models with humans, a single parameter in each model was fit to human data: the boundary value above which the change was taken to be an increase (i.e., “more”) or decrease/no change (i.e., “less”/“neutral”). The “less” and “neutral” categories were combined because they were much smaller than the “more” category in human data. The optimal value for this parameter was found by sweeping through the range (−1, 1) and finding the value that resulted in the highest number of matching responses with the 631 and 1,966 questions. Further, a second boundary was introduced to capture the “neutral” responses (it was initialized where the first boundary ended).

##### Results

The three approaches to measuring the predictions of the CEREBRA model, i.e., the context effect of the rest of the sentence, the context effect of the entire sentence, and the context effect of the word in different contexts, were implemented and fit to human data using two-boundary model fitting. The three approaches produced remarkably similar results. Furthermore, the first two approaches achieved slightly better results than the third one (by 2%).

The match results for each set of questions are presented in Tables 4A, 5A and the statistical significance in Tables 4B, 5B, respectively. Table 4A shows that CEREBRA Approaches 1 and 2 match human responses in 77% and for Approach 3 in 75% of the questions, while the chance level is 68% - which is equivalent to always guessing “more”, i.e., the largest category of human responses. Similarly, Table 5A shows that CEREBRA Approaches 1 and 2 match human responses in 55% and for Approach 3 in 54% of the questions, while the chance level is 45% (i.e., always guessing “more”). The differences shown in Tables 4B, 5B, include the means and variances of the CEREBRA change models and the chance model for each subject, and the *p*-values of the Student *t*-test, revealing that the differences are highly statistically significant for all of the 8 subjects for the three approaches shown. These results indicate that the changes in word meanings due to sentence context (observed in the fMRI and interpreted through semantic feature representations) are real and meaningful to the subjects.

**Table 4:** CEREBRA match results and statistical significance compared with human judgements across sentences where all four subjects agreed.

**(A) Matching CEREBRA predictions for approaches one to three and chance with human data**											
**All four participants average agreement (3 ratings)**											
**Ratings**	**Human**		**Cerebra#1**		**Cerebra#2**		**Cerebra#3**			**Chance**	
−1	190		145		149		134			1	
0	15		0		0		0			0	
1	426		341		336		339			426	
**Total**	631		486		485		473			427	
**Average**			77%		77%		75%			68%	
**(B) Statistical analyses for CEREBRA approaches and chance**											
**Subjects**	**Chance**		**Cerebra #1**		**Cerebra #2**		**Cerebra #3**		***P*** **-value**	***P*** **-value**	***P*** **-value**
	**Mean**	**Var**	**Mean**	**Var**	**Mean**	**Var**	**Mean**	**Var**	**Cerebra #1**	**Cerebra #2**	**Cerebra #3**
S1	427	0.91	486	46.74	486	56.42	466	152.98	5.42e-32	1.66e-30	1.17e-16
S2	427	1.10	481	32.62	480	21.54	466	105.61	1.67e-33	2.02e-36	2.30e-19
S3	426	0.57	486	42.58	485	37.85	480	39.29	6.50e-33	1.65e-33	6.22e-32
S4	427	1.69	486	21.95	486	27.73	481	32.62	1.46e-37	6.25e-36	2.55e-33
S5	427	1.71	490	57.00	488	57.09	470	89.12	3.80e-31	7.56e-31	8.82e-22
S6	427	2.87	486	44.06	484	34.04	469	80.66	6.59e-32	3.17e-33	6.29e-22
S7	427	2.77	489	24.77	489	21.21	483	54.05	3.09e-37	2.93e-38	1.62e-29
S8	427	1.67	480	75.78	480	54.22	471	92.68	1.82e-26	4.62e-29	5.56e-22

***(A)** The table shows the average agreement of the 20 repetitions across all 8 fMRI subjects. CEREBRA Approaches 1 and 2 agree with human responses 77%, CEREBRA Approach 3 agrees 75%, when the chance level is 68%. **(B)** The table shows the means and variances of the CEREBRA change models and the chance model for each subject, and the p-values of the Student t-test, revealing that the differences are highly significant. Comparison agreement with human judgements where all four subjects agreed*.

**Table 5:** CEREBRA match results and statistical significance compared with human judgements across sentences where at least three of the four subjects agreed.

**(A) Matching CEREBRA predictions for approaches one to three and chance with human data**											
**Three of four participants average agreement (3 ratings)**											
**Ratings**					**Human**		**Cerebra#1**		**Cerebra#2**	**Cerebra#3**	**Chance**
−1					618		478		484	463	8
0					456		2		2	3	0
1					892		608		599	587	886
**Total**					1966		1088		1085	1053	894
**Average**							55%		55%	54%	45%
**Statistical analyses for CEREBRA approaches and chance**											
**Subjects**	**Chance**		**Cerebra #1**		**Cerebra #2**		**Cerebra #3**		***P*** **-value**	***P*** **-value**	***P*** **-value**
	**Mean**	**Var**	**Mean**	**Var**	**Mean**	**Var**	**Mean**	**Var**	**Cerebra #1**	**Cerebra #2**	**Cerebra #3**
S1	894	6.01	1,082.5	149.0	1083	131.32	1,033	707.25	2.94E-41	2.99E-42	3.92E-24
S2	894	7.21	1,076.8	199.0	1073	128.31	1,035	233.91	2.15E-38	1.80E-41	6.10E-33
S3	894	11.52	1,089.4	186.6	1086	166.91	1,063	224.41	8.89E-40	2.48E-40	5.22E-36
S4	894	7.21	1,086.7	39.0	1087	36.64	1,077	94.79	1.51E-51	5.06E-52	3.89E-44
S5	895	12.03	1,099.1	183.8	1097	157.71	1,048	252.79	1.19E-40	1.12E-41	1.83E-33
S6	894	4.62	1,088.0	179.5	1082	161.88	1,048	205.82	2.64E-40	1.24E-40	1.73E-35
S7	895	7.21	1,097.6	64.1	1096	41.73	1,075	216.77	8.52E-49	8.54E-52	1.65E-37
S8	894	2.52	1,079.6	229.6	1077	129.91	1,039	366.06	1.09E-37	5.10E-42	6.10E-30

***(A)** The table shows the average agreement of the 20 repetitions across all 8 fMRI subjects. CEREBRA Approaches 1 and 2 agree with human responses 55%, CEREBRA Approach 3 agrees 54%, when the chance level is 45%. **(B)** The table shows the means and variances of the CEREBRA change models and the chance model for each subject, and the p-values of the Student t-test, revealing that the differences are highly significant. Comparison agreement with human judgements where at least three of the four subjects agreed*.

## Discussion and Future Work

Word meanings have long been known to change in the context of a sentence (Harris, 1954; Firth, 1957). However, this research is novel in two respects: by using CARs, i.e., brain-based word representations (instead of text-based word representations), and by using fMRI observations (that include word meanings) to constrain the CARs. Despite recent success in text-based semantic modeling and multimodal word representations, there is still a great deal of disagreement about how semantic knowledge is represented in the brain and whether these models correlate with actual brain representations. In contrast, the semantic model used in this research is built from interpretable features, supported by substantial evidence on how humans acquire and learn concepts through different modalities, spans many aspects of experience comprehensively, and thus provide a way to understand the semantic space of the brain (Binder et al., 2009, 2016; Binder and Desai, 2011; Binder, 2016). Therefore, understanding how grounded and embodied word meanings change under the context of a sentence may be a useful starting point for studying the mental lexicon.

The CEREBRA model built on this theory generates good interpretations of word meanings especially considering that the dataset was limited and was not originally designed to address the dynamic effects of meaning. It would be interesting to replicate the studies on a more extensive data set. A fully balanced stimuli including sentences with identical contexts (e.g., *The yellow bird flew over the field* vs. *The yellow plane flew over the field*) and contrasting contexts (e.g., *The aggressive dog chased the boy* vs. *The friendly dog chased the boy*), could help characterize the effects in detail. The context-based changes should be even stronger, and it should be possible to uncover more refined effects.

Similarly, it would be desirable to extend the fMRI data with images for individual words. The CEREBRA process of mapping semantic CARs to SynthWords and further to sentence fMRI refines the synthetic representations by removing noise. However, such representations blend together the meanings of many words in many sentences. Hence, by acquiring actual word fMRI, the observed effects should become sharper.

Given how noisy human response data is, the 7%, 9%, and 10% differences between CEREBRA and chance are strong results. Human raters do not often agree; their judgement is influenced by experience and uncertainty, in addition to factors such as age, language, and education. Inter-rater reliability could be improved by training the raters so that they become comfortable with the concepts of “generic meaning” and “variable meanings”. It may also be possible to design the questions such that they allow comparing alternatives, which may be easier for the participants to respond.

CAR theory has already been validated in many studies (Fernandino et al., 2015; Anderson et al., 2016, 2018; Binder et al., 2016). Therefore, this research took it as a starting point in building CEREBRA. However, whereas the original CAR concerns static representations, CEREBRA extends it to dynamic representations, and shows how they can change based on context.

One disadvantage of CEREBRA is that it is expensive to collect fMRI patterns and human ratings at a massive scale compared to running a statistical algorithm on a data repository. Furthermore, any changes to the CARs (e.g., adding features) would require new data to be collected. However, such data provides a grounding to neural processes and behavior that does not exist with statistical approaches. This difference becomes evident when the CAR semantic model is compared to approaches such as Conceptual Spaces (Gardenfors, 2004; Bechberger and Kühnberger, 2019; CS), and distributional semantic models (Landauer and Dumais, 1997; Burgess, 1998; Mitchell and Lapata, 2010; Silberer and Lapata, 2012, 2014; Anderson et al., 2013; Mikolov et al., 2013; Bruni et al., 2014; DSMs). Both, CAR theory and CS characterize concepts with a list of features or dimensions as the building blocks. Importantly, they include similar dimensions (i.e., weight, temperature, brightness) and some of those dimensions are part of a larger domain (e.g., color) or a process (e.g., visual system). The CAR theory provides a set of primitive features for the analysis of conceptual content in terms of neural processes (grounded in perception and action). Instead, the CS framework suggests a set of “quality” dimensions as relations that represent cognitive similarities between stimuli (observations or instances of concepts).

Compared to DSM, the CAR theory is a brain-based semantic feature representation where people weigh concept features differently based on context. DSMs are not grounded on perception and action mechanisms (i.e., words are defined by other words). They reflect semantic knowledge acquired through a lifetime of linguistic experience, found in the corpus used for training the model, based on statistical co-occurrence, and do not provide precise information about the experienced features of the concept itself (Anderson et al., 2016). They are models of word meaning as they are in the text (Sahlgren, 2008). CEREBRA takes good advantage of such a grounding by representing word meanings as they are “in the head”. The CAR features relate semantic content to neural activity, which can then be verified with fMRI.

## Conclusion

The CEREBRA model was constructed to test the hypothesis that word meanings adapt dynamically based on context. The results support three conclusions: (1) context-dependent meaning representations are embedded in the fMRI sentences, (2) they can be characterized using CARs together with the CEREBRA model, and (3) the attribute weighting changes are real and meaningful to human subjects. Thus, CEREBRA opens the door for cognitive scientists to achieve better understanding and form new hypotheses about how semantic knowledge is represented in the brain. Overall, this research is expected to contribute to the development of a unified theory of concepts, the organization of the semantic space, and the processes involved in word meaning representation. CEREBRA promotes further research on issues such as how words can be related thematically, how concepts can be combined, how word meaning can be formed, and how different individuals perceive the world (i.e., cultural differences), thus advancing the understanding of grounded representations in the mental lexicon.

## Data Availability Statement

The data analyzed in this study is subject to the following licenses/restrictions. The original and new CAR collections are available. The fMRI data is not public. Requests to access the available datasets should be directed to NA-C, naguirre@cs.utexas.edu. For the fRMI data, contact Jeffrey Binder at Medical College of Winsconsin.

## Ethics Statement

The studies involving human participants were reviewed and approved by University of Texas Institutional Review Board (2018-08-0114). The patients/participants provided their written informed consent to participate in this study.

## Author Contributions

All authors listed have made a substantial, direct, and intellectual contribution to the work and approved it for publication.

## Funding

This work was supported in part by IARPA-FA8650-14-C-7357 and by NIH 1U01DC014922 grants.

## Conflict of Interest

The authors declare that the research was conducted in the absence of any commercial or financial relationships that could be construed as a potential conflict of interest.

## Publisher's Note

All claims expressed in this article are solely those of the authors and do not necessarily represent those of their affiliated organizations, or those of the publisher, the editors and the reviewers. Any product that may be evaluated in this article, or claim that may be made by its manufacturer, is not guaranteed or endorsed by the publisher.

## Footnotes

^1^The FGREP mechanism is similar to a three-layer back-propagation neural network. It follows the same dynamics to learn the task by adapting the connection weights. However, in order to develop meaningful word representations, the error signal is extended to the input layer.
